# Randomly weighted receptor inputs can explain the large diversity of colour-coding neurons in the bee visual system

**DOI:** 10.1038/s41598-019-44375-0

**Published:** 2019-06-06

**Authors:** Vera Vasas, Fei Peng, HaDi MaBouDi, Lars Chittka

**Affiliations:** 1 Bee Sensory and Behavioural Ecology Lab, Department of Experimental and Biological Psychology, School of Biological and Chemical Sciences, Queen Mary University of London, London, UK; 2 Department of Psychology, School of Public Health, Southern Medical University, 1838 Guangzhou Road, Guangzhou, 510515 Guangdong China; 3 Wissenschaftskolleg zu Berlin, Institute for Advanced Study, Wallotstrasse 19, D-14193 Berlin, Germany

**Keywords:** Computational models, Neural circuits, Colour vision

## Abstract

True colour vision requires comparing the responses of different spectral classes of photoreceptors. In insects, there is a wealth of data available on the physiology of photoreceptors and on colour-dependent behaviour, but less is known about the neural mechanisms that link the two. The available information in bees indicates a diversity of colour opponent neurons in the visual optic ganglia that significantly exceeds that known in humans and other primates. Here, we present a simple mathematical model for colour processing in the optic lobes of bees to explore how this diversity might arise. We found that the model can reproduce the physiological spectral tuning curves of the 22 neurons that have been described so far. Moreover, the distribution of the presynaptic weights in the model suggests that colour-coding neurons are likely to be wired up to the receptor inputs randomly. The perceptual distances in our random synaptic weight model are in agreement with behavioural observations. Our results support the idea that the insect nervous system might adopt partially random wiring of neurons for colour processing.

## Introduction

Bees are extensively studied for their colour vision. Behavioural^1–3^ and electrophysiological^4–6^ work has established that the vision of most species of bees is trichromatic, receiving input from three spectral types of photoreceptors whose sensitivity peaks in the UV (for honeybees, λ_max_ ≈ 344 nm), blue (for honeybees, λ_max_ ≈ 436 nm) and green (for honeybees, λ_max_ ≈ 544 nm) parts of the spectrum. These wavelength positions are close to the theoretical optimum for coding flower colours^7^. As a result, bees have excellent colour discrimination abilities – an essential skill for a pollinator that relies on colour vision to recognise flowers and forage among various food resources^8,9^. The trichromatic vision of bees follows the Grassmannian colour mixture and matching laws^1^, which were established first in humans. Bees show similar effects of simultaneous and sequential colour contrast as humans do^10,11^, and also have colour constancy, allowing them to recognise flower colours even in the face of profound spectral changes in ambient light^12–14^.

Despite these similarities of the human and the bee trichromatic system, there are also some differences that extend beyond the fact that the visual spectrum is shifted, in bees, to shorter wavelengths in its entirety. Profound differences, for example, are apparent in the neural processes that evaluate the signals from the three colour receptors types. In humans and other trichromatic primates, opponent interactions in the retina occur between the long-wavelength-sensitive (L) and the medium-wavelength-sensitive (M) cones, and between the short-wavelength-sensitive (S) cones and some combination of the M and L cones^15,16^. Cone opponent responses arise at the level of retinal ganglion cells after the first postreceptoral synapse. These cells display a characteristic spatial antagonism: they receive antagonistic input from their centre and their surround. Provided that the centre is small enough, the spatial structure in itself yields cone opponent responses. Accordingly, the ‘random wiring hypothesis’ suggests that the L-M opponency in midget ganglion cells appears without cone-specific wiring, by comparing the responses of a very small centre (typically one cone) to its larger, randomly assembled surround^17–20^. Thus, randomness might be an important organizing principle of colour processing in humans, although the neural circuits also make use of complex cone-specific wiring mechanisms and a diversity of ganglion cell types^19,21^. The spatial antagonism between the centre and surround in the receptive fields of retinal ganglion cells and a set of additional modulatory processes ensure that there are two classes of cone opponent processes, with predictable inputs from the three spectral receptor types, that form the foundation of colour opponency and colour perception as measurable in psychophysical experiments^15,22–27^.

In bees, however, there is no known spatial antagonism in the receptive fields of colour coding neurons^28–30^, and the early processing of chromatic information is separate from the evaluation of achromatic information^31–34^. Here we propose that this fundamental difference has important implications for the entirety of colour processing and perception. Early theoretical work assumed deterministic wiring from receptors to colour sensitive neurons, and indicated that two spectrally antagonistic neuron types might also explain colour discrimination in bees^35,36^ (perhaps inspired by the colour opponency mechanisms in humans). Empirical electrophysiological work in honeybees and bumblebees, however, identified a much larger diversity of colour-sensitive neurons than the two classes found in humans^28–30,33,34,37–42^. If we make no assumptions about deterministic wiring, given three spectral receptor signals (S, M, L for short-, medium- and long-wavelength-sensitive receptors), and the fact that each can provide either excitatory or inhibitory input to a neuron or can be ignored, 26 different categories of inputs are possible. Of these, six will receive input from one receptor type (S+; M+; L+ or S−; M−; L−); eight will add up input from multiple types (S+ M+; S+ L+; M+ L+; S+ M+ L+; S− M−; S− L−; M− L−; S− M− L−) and twelve different combinations will be colour opponent neurons receiving spectrally antagonistic inputs (S+ M−; S− M+; S+ L−; S− L+, M+ L−; M− L+; S+ M− L−; S− M+ L+; S− M+ L−; S+ M− L+; S− M− L+; S+ M+ L−). From these 26 theoretically possible categories, only three have not (yet) been found in either the medulla or the lobula of honeybees and bumblebees^9,43^. Moreover, as noted by Kien & Menzel^28,30^ and Hertel^29^, the diversity of response patterns even within each category suggests marked differences in wiring patterns.

This diversity is at odds with the notion that colour-coding neurons in bees are wired deterministically. Instead, we put forward a simpler model that assumes random inputs from receptors to colour-coding neurons (as in the ‘random wiring’ model discussed above), but without spatial antagonism in the neurons’ receptive fields. Such random wiring has the implicit simplicity that during development, growing neurites from colour coding neurons need not find specific cells (or vice versa). Instead, *any* connection between a sensory input pathway and the colour-coding cell will do, and as the neurons bringing in colour information may be both excitatory or inhibitory, colour specific responses and opponency will arise by mere stochastic processes. In fact, such stochastic processes can drive cell type specification in insects^44,45^, and random connectivity has been described in the insect olfactory system (between olfactory projection neurons and mushroom body cells in the fly)^46,47^ and in human motor control^48^. Contrary to intuition, advances in machine learning have shown that such randomness can lead to efficient representations in neural networks^49,50^.

We ask if the empirically observed large variety of colour sensitive responses in bees can be explained by the assumption that colour-coding cells receive randomly weighted input from the different spectral receptor types. We outline a biologically plausible neural model of colour processing in bees and discuss some important implications of random wiring for colour perception in bees. We conclude that (1) functional diversity within one class of morphological colour-coding neuron type is enough to generate all observed spectral response diversity, including non-colour-opponent and colour opponent neurons alike, as long as we (2) assume random synaptic weights from presynaptic neurons and (3) allow for different baseline firing rates and for non-linear activation functions.

## Results

### Neural network model for colour coding

**Figure 1:**
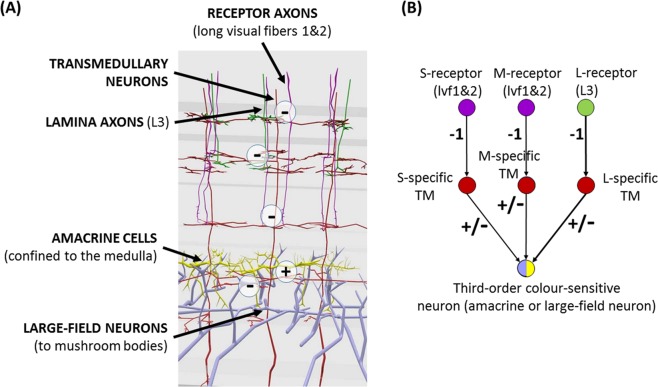
Neuron morphology and model structure. (**A**) The second neuropil, the medulla, is responsible for the early processing of colour information. Receptor axons (long visual fibres, lvf 1–2) of short (S)- and medium-wavelength-sensitive (M) receptors and lamina axons (L3), conveying long-wavelength information (L), form inhibitory synapses on transmedullary (TM) neurons in the first two layers of the medulla. (**B**) In our model, the presynaptic weights to TM cells (*v*_S_, *v*_M_, *v*_L_) are set to −1. Transmedullary neurons, in turn, either excite or inhibit third-order cells (amacrine and/or large field neurons) in the serpentine layer (layers 4–5). See http://chittkalab.sbcs.qmul.ac.uk/VeraPub/neurons3d/index.html for an interactive 3D representation of the neuronal wiring. The model presented in this paper analyses in detail the spectral profiles of third-order colour sensitive neurons.

We compiled the available information on neuron morphology^38,51^, electrophysiology^28–30,38^ and immunohistochemistry^38,52–54^ relevant to colour sensitive neurons in bees (*Apis mellifera* and *Bombus impatiens*) and deduced a simple model of circuitry that is in line with our current knowledge (Fig. 1). The initial stages of colour processing take place in the medulla, the second and largest of the three optic ganglia of the bee^38^. Short (S)- and medium-wavelength-sensitive (M) photoreceptor axons project here directly. The information from long-wavelength-sensitive (L) receptors reaches the medulla indirectly, through monopolar cells in the lamina (the first optic ganglion), which in turn send projections to the outer layers of the medulla (Fig. 1A)^51^.

Immunohistochemistry identified histamine as the neurotransmitter in these layers^38,52,54^, which is considered to be predominantly an inhibitory neural transmitter in insect brains across taxa^55^. Thus, we put forward the hypothesis that the inputs make inhibitory connections to the columnar narrow-field neurons called transmedullary (TM) cells that originate here. These neurons have high baseline activities and respond to stimulation by decreasing their firing rates^38^. We modelled the simplest case, where one type of receptor connects to one type of transmedullary cell (Fig. 1B), thus three distinct types of transmedullary cells respond to either short-, medium- or long-wavelength stimulation; such neurons thus have spectral sensitivities that match the sensitivity of receptors (Supplementary Fig. S1; see Discussion for the alternative possibility of lateral connections in this layer). A more broad-band sensitivity emerges when transmedullary cells make connections with multiple types of input fibres (Supplementary Fig. S1). This characterization matches electrophysiological observations about the response properties of transmedullary cells (i.e., either receptor-specific or broad-band inhibitory response to light)^38^.

**Figure 2:**
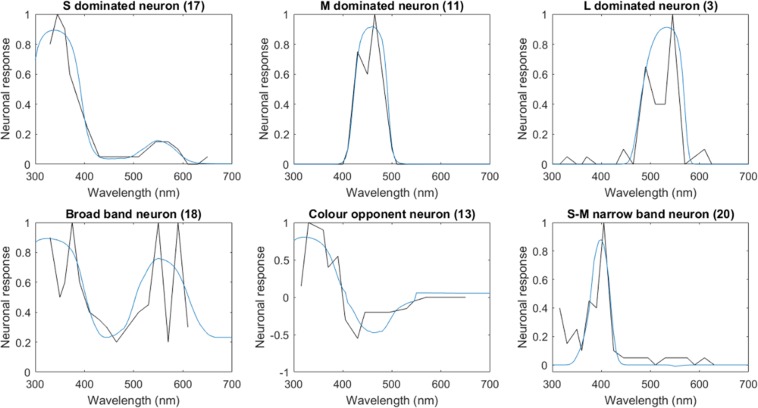
Spectral tuning curves of third-order neurons. All figures show the normalised (dimensionless) change in neuron response as a function of the wavelength of monochromatic lights of equal intensity. Black lines depict the empirically measured spectral tuning curves from^28,30^; blue lines indicate the best fit curves generated by the model. Narrow-band, broad-band and colour-opponent neurons can be generated by varying the presynaptic weights and threshold properties of the activation function of the neuron. For parameter estimates and more examples, see Supplementary Fig. S2 and Table S1.

We suggest that transmedullary cells, in turn, connect to either third-order amacrine or to third-order large field cells, which run perpendicular to the transmedullary cells in the middle layers of the medulla (the serpentine layer). Judging by the diversity of neurotransmitters in the serpentine layer^38,53^, these connections can be both excitatory or inhibitory. Amacrine cells do not have projections outside the medulla, while large field neurons project to the principal learning centres of the bee brain^56^, the mushroom bodies. In our model, and in line with the conclusion of experimental studies^38^, these third-order neurons constitute the first layer where colour information can be extracted through comparing the responses of different spectral types of receptors. We make no assumption about the synaptic weights between second-order transmedullary cells and third-order cells. The activation function of the modelled neurons is defined in a way that the neuron does not respond at all below a certain threshold stimulation, and responds maximally above another threshold, equivalent to an S-shaped rectified linear unit (SReLU) in psychophysics^57^. Most importantly, the morphology assumed for all third-order cells is the same – following the empirical observations on amacrine and large field neurons in the bee medulla^38^ – but each cell is characterized by a different response threshold and different, randomly distributed presynaptic input weights.

### A simple model can reproduce previously measured spectral response curves with one morphological neuron type

The papers by Kien and Menzel^28,30^ are unique in the literature to report complete spectral tuning curves of colour-sensitive neurons of bees. They characterized the responses of 22 higher-order colour-sensitive neurons to a series of monochromatic lights (Supplementary Fig. S2). All tested neurons had large receptive fields, showing that they were either amacrine, large-field or other higher order neurons, not transmedullary cells. Partial measurements^28–30^ and qualitatively reported responses^29,38^ do not allow predictions on exact presynaptic inputs; however, they provide further evidence for the diversity of the colour sensitivity profiles of optic lobe neurons.

We tested the ability of our simple neural network model to reproduce the spectral tuning curves reported by Kien and Menzel^28,30^. These recordings describe three colour opponent cells, which received excitatory input from the UV receptors and inhibitory input from the blue and green receptors (labelled as 13, 14, 16 in our analysis), and 19 neurons that displayed non-colour opponent, but colour-dependent responses. The recordings were made from both the medulla and the lobula, and the majority of the electrodes have unknown locations; by aiming to reproduce all of these curves, we subjected our model to a stronger test than strictly necessary. We used a gradient descent algorithm to find model parameters (presynaptic weights and threshold parameters of the activation function) that generated the most similar curve to the empirical one in terms of least squared differences (see Methods). We found that for each and every empirical tuning curve, there exists a parameter combination that can explain the neuron’s response in terms of deviation from the baseline activity (the coefficient of determination R^2^ = 0.77–0.99, or 77–99% variance explained; see Supplementary Table S1); in other words, the model neuron’s response profile fits the shape of the empirical neuron’s profile (Fig. 2, and Supplementary Fig. S2). Thus, our results suggest the diversity of colour responses in bees does not need to rely on multiple neuron types with diverse morphologies. Instead, a variety of presynaptic input weights and variable neuronal firing properties could be responsible for the observed functional diversity.

### The estimated weight of photoreceptor inputs to colour-sensitive neurons in the model follows a random distribution

**Figure 3:**
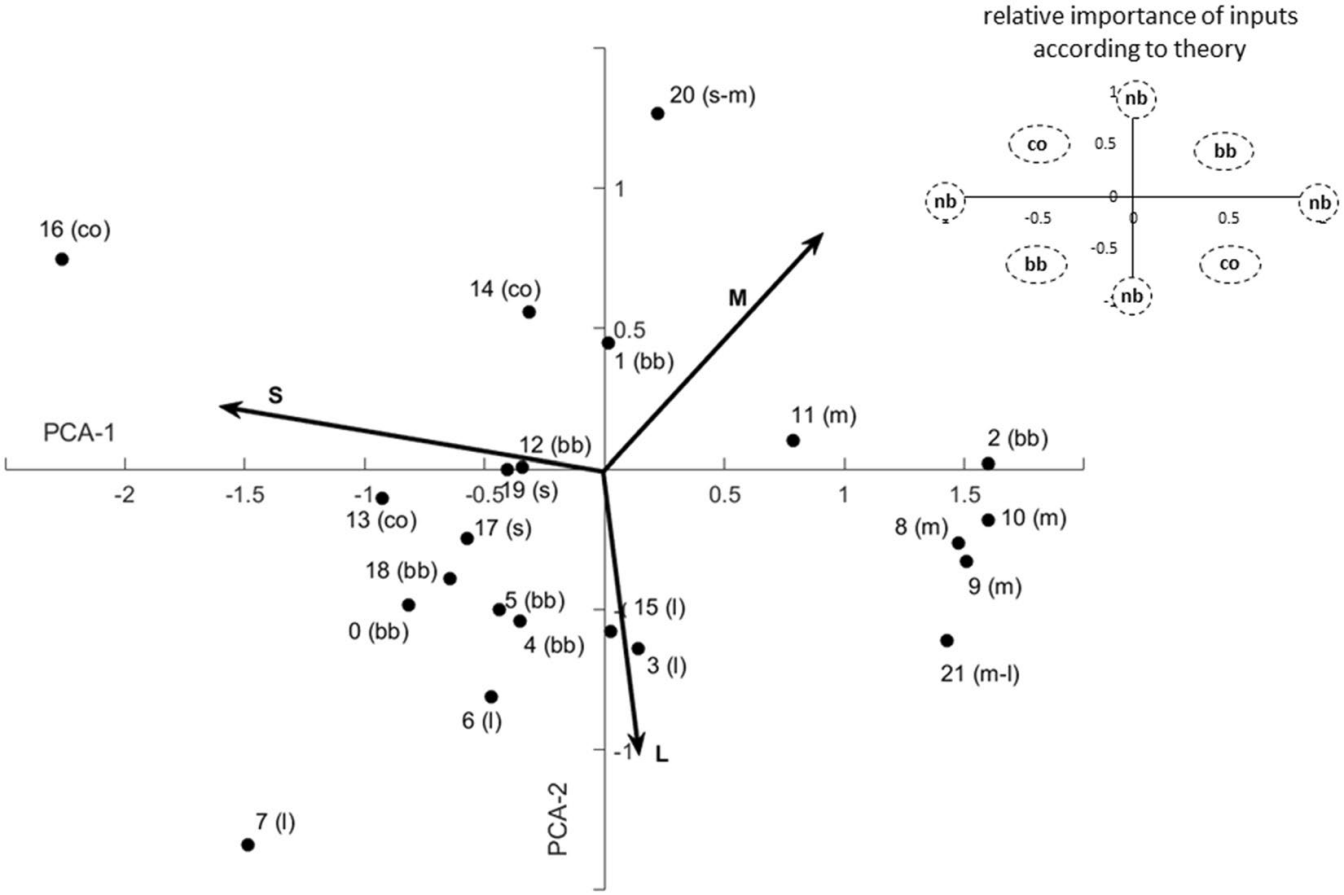
Presynaptic weight distribution proposed by the model for the empirical data. The x and y axes indicate the first two axes proposed by PCA for the three-dimensional distribution of weights and explain 95.2% of the variation. The arrows indicate individual excitatory input from the short (S)-, medium (M)- and long (L)-wavelength-sensitive receptors. Each data point represents the presynaptic weights the model proposed for one spectral tuning curve. Intuition suggests that broad-band neurons should receive similar input from all types of receptors; colour opponent neurons should be excited by one receptor type and inhibited by others; and so on (see Inset in the top right corner), but the weights in our model do not cluster according to the response characteristics of the neurons. Instead, they are randomly distributed along a continuum. nb: narrow-band, bb: broad-band, co: colour-opponent, l: L-dominated narrow-band, m: M-dominated narrow-band, s: S-dominated narrow-band, s-m narrow-band with the peak between S and M peak sensitivities, m-l: narrow-band with the peak between M and L sensitivities.

Next, we analysed the shape of the activation function and the presynaptic weights our model proposed for the 22 neurons. Kien and Menzel^28,30^ used the following categories to describe their colour sensitive neurons: narrow-band neurons that respond only to a small wavelength range of light, broad-band neurons that respond to light across the full visual spectrum, and colour opponent neurons that respond to stimuli either with excitation or inhibition, depending on the wavelength of the light (Fig. 2). Our model, on the other hand, supports Hertel^29^, who came to the conclusion that transitional forms between the categories are abundant and thus stated that ‘there is no evidence that these classes represent actual basic mechanisms for the processing of visual signals in the bee’. Our model suggests that nearly all neurons (20 out of 22) receive substantial input (>10% relative weight) from more than one receptor type, and the majority of neurons (15 out of 22) appears to receive substantial input from all receptor types (see Supplementary Table S1). Interestingly, spectral tuning curves narrower that the original receptor sensitivities result from steep neuronal activation functions, and not from inhibitory inputs per se, as suggested in earlier studies^28–30^. The visualisation of the first two principal components of the presynaptic weight distribution of the model suggests that the weights are scattered along a continuum (Fig. 3, and Supplementary Fig. S5). We performed a clustering analysis with Dirichlet process Gaussian mixture models^58^ (see Methods) to estimate the optimal cluster number. The model consistently outputs (100 repeated runs with different random seeds) only one cluster, confirming the conclusion based on the visual inspection of the data. Thus, our evidence points to the possibility that colour sensitive neurons in the bee brain wire up randomly to the photoreceptor inputs, and that this randomness is the reason for the large diversity of colour-sensitive responses.

### Spectral tuning curves generated by the model for third order neurons receiving randomly weighted inputs

**Figure 4:**
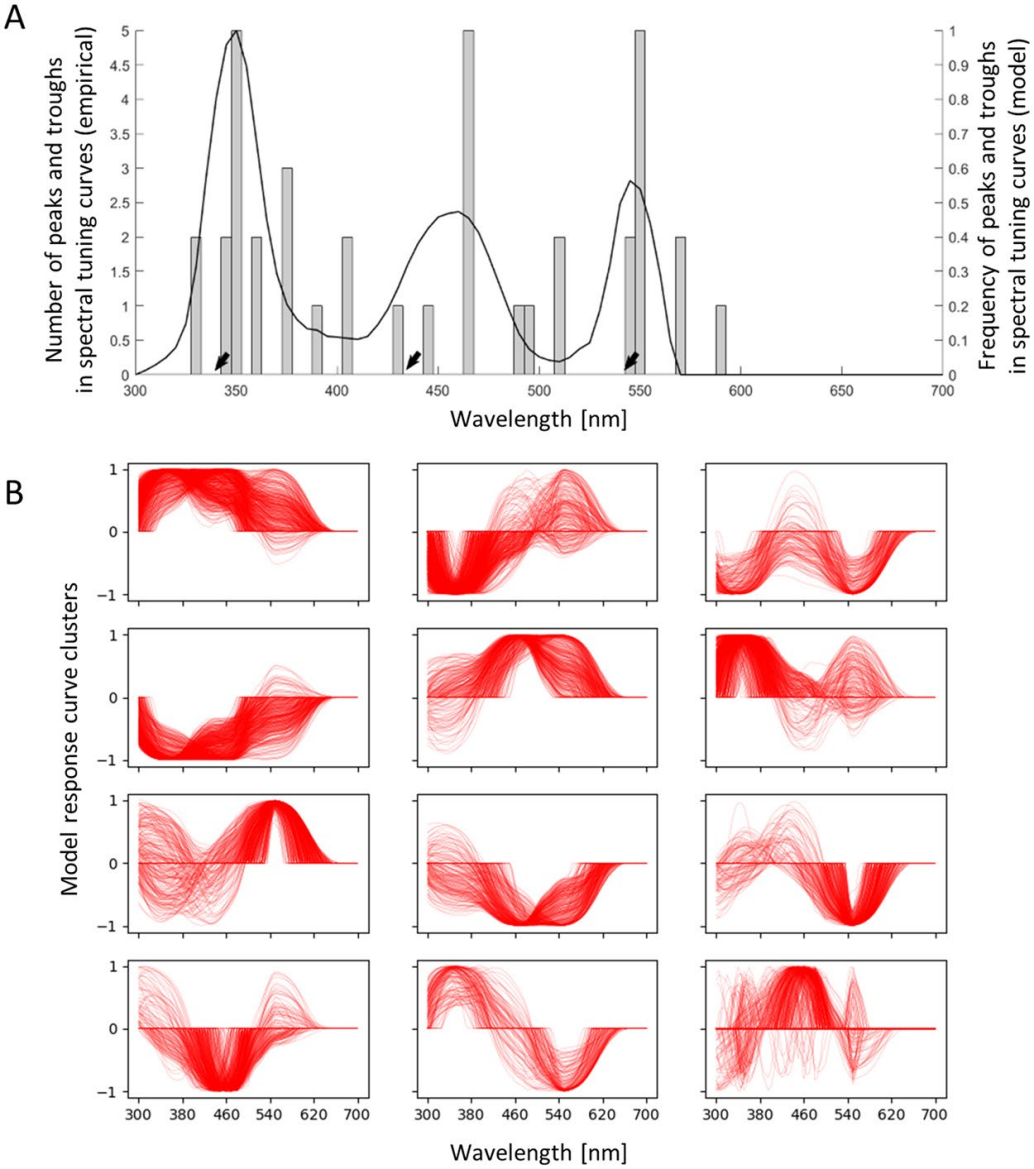
Comparisons between simulated neuronal responses and empirical data. (**A**) The distribution of peaks and troughs in the spectral tuning curves generated by the model follows a similar pattern as the empirical measurements. Grey bars: the peak frequency of the 22 empirically recorded neurons, black line: peak frequency at all the wavelengths from the library of 5500 simulated neuronal responses. The three black arrows indicate where the receptors themselves are maximally sensitive. Note that the wavelengths of maximal receptor sensitivity do not line up fully with the distribution of peaks and troughs in higher-order neurons, either given by the empirical neurons or the model. (**B**) Through Dirichlet process Gaussian mixture modelling, we show that the 5500 neuronal responses could be best described by 9–14 clusters (11.08 ± 1.03 s.d.;100 repeated runs). (**C**) Spectral response curves grouped into 12 clusters are plotted as 12 panels. These clusters describe the spectral response types that appear in our model when neurons are randomly wired up to photoreceptors. Despite the existence of basic types, there is considerable variation within each type. See also Supplementary Fig. S6 for similar results from time-series K-Means clustering with silhouette analysis.

In the following analysis, we directly addressed this diversity of colour-sensitive neurons. We compiled a library of neuronal responses that can be produced by our model. While the diversity is indeed large, not all spectral tuning curves are possible. Notably, the distribution of the neurons’ maximal (peaks) and minimal (troughs) response sensitivities follows a similar pattern as the empirical data, with peaks/troughs occurring with high probability near the maximal sensitivities of the S and the L photoreceptors (around 344 nm and 544 nm), with a low probability at the peak sensitivity of the M photoreceptor (around 436 nm), but with a peak where the M and L photoreceptor sensitivity curves overlap maximally (around 460–470 nm; Fig. 4A). We performed both Dirichlet process Gaussian mixture modelling^58^, as well as time-series K-Means clustering^59^ with silhouette analysis^60^ to examine the clusters in the library of model neuronal responses. The Dirichlet process Gaussian mixture models show that on average (100 repeated runs), 9–14 clusters are optimal for the given simulated neuronal response library, indicating the existence of approximately 12, more or less distinct spectral response types (Fig. 4B). The time-series K-Means clustering yields consistent results (8–16 clusters have relatively high silhouette coefficients; Supplementary Fig. S6). Thus, our analysis shows that randomly wired neurons do not produce random responses, instead, the theoretically possible spectral tuning curves cluster into 8–16 response categories (although large variations within categories are possible).

### Perceptual distances as suggested by the model agree with behavioural observations

The structure in the shapes of the spectral tuning curves has important implications for how stimuli appear in the perceptual space of bees. Behavioural experiments have established that the bees’ ability to distinguish monochromatic light sources depends on the wavelength region within their visual spectrum: discrimination is best around 400 and 500 nm^2^. Another set of experiments^61–63^ showed that the success of discrimination on a grey background scales non-linearly (probably following a sigmoidal function^64^) with colour difference, and that bees reach perfect discrimination at smaller colour distances for shades of blue vs. shades of yellow.

**Figure 5:**
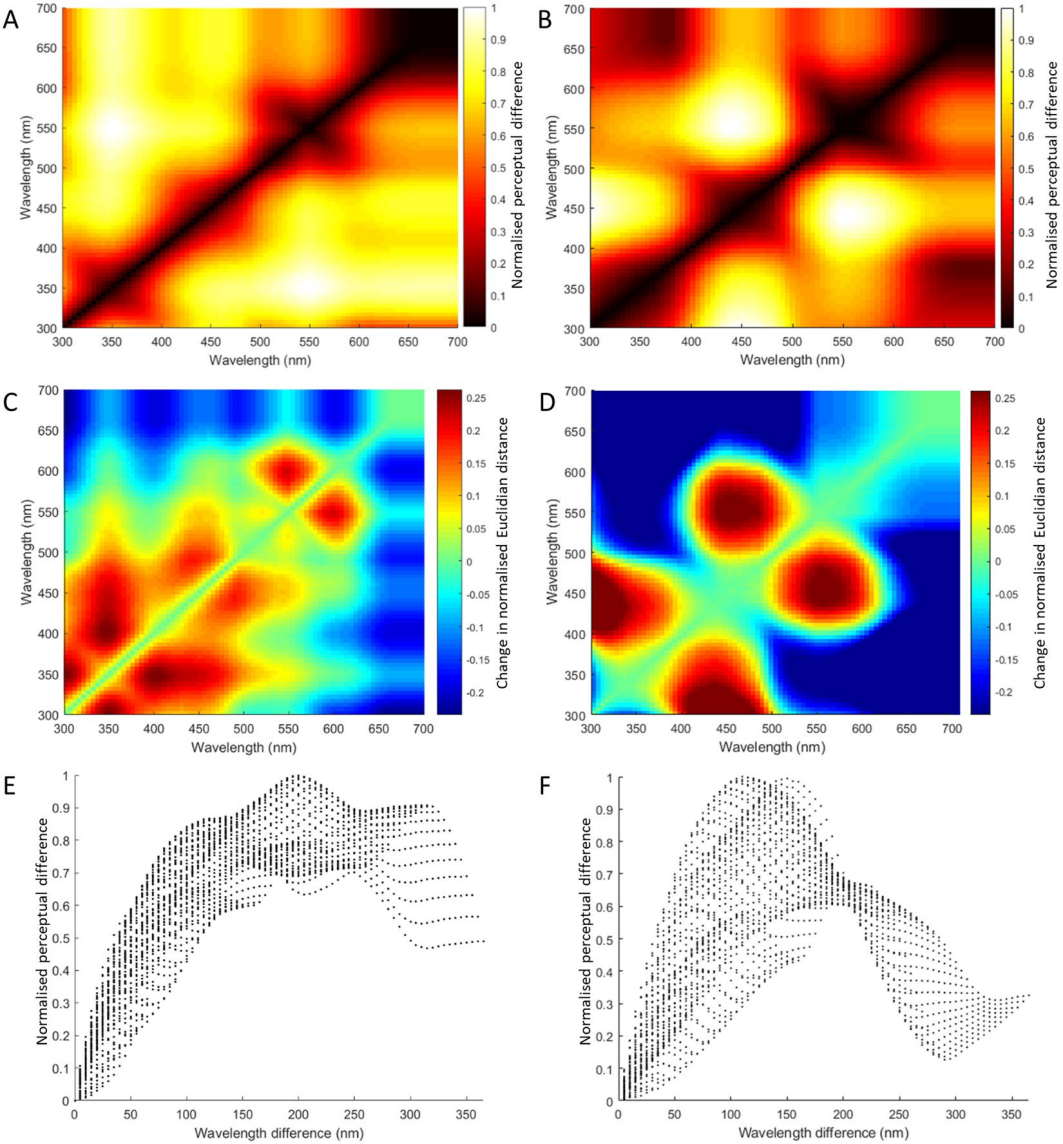
Perceptual differences predicted by our randomly wired model are in agreement with behavioural observations. We define the perceptual difference between two monochromatic stimuli as the Euclidean distance between the responses of all neurons to them. (**A**) Perceptual differences across all combinations of monochromatic stimuli in our random model. The colour of each point in the heatmap indicates the normalised perceptual difference between two monochromatic stimuli whose wavelengths are given on the x and y axes. Note that perceptual differences are larger in the blue than in the yellow region of the spectrum, and that they are larger between the peak sensitivities of receptors (~400 and ~500 nm) than at the peaks themselves. (**B**) Perceptual differences across all combinations of monochromatic stimuli in a regularly wired model. The colour of each point in the heatmap indicates the normalised perceptual difference between two monochromatic stimuli whose wavelengths are given on the x and y axes. (**C**) Changes in Euclidean distances between receptor responses and randomly wired third-order neurons across all combinations of monochromatic stimuli. Neural processing downstream of the receptor level enhances the perceptual differences between similar colours, especially in the UV and blue region of the spectrum. (**D**) Changes in Euclidean distances between receptor responses and, this time, regularly wired colour-opponent neurons, across all combinations of monochromatic stimuli. In this case, neural processing downstream of the receptor level would enhance the perceptual differences in the areas where the sensitivities of the receptors overlap. (**E**) In the random model, perceptual differences scale as a non-linear, approximately monotonic function of wavelength difference, across all combinations of monochromatic wavelengths. Monotonic scaling ensures that perceptual distances reflect dissimilarity in stimuli. (**F**) In the regularly wired model, perceptual differences do not scale with difference in wavelengths, and accordingly, do not reflect dissimilarity in stimuli. See also Supplementary Fig. S7.

In this section, we analysed the ensemble response of our library of 5500 third order neurons (see Methods) to monochromatic lights. We defined the perceptual distance between two monochromatic stimuli as the Euclidean distance between all colour neuron responses to the stimuli. First of all, the perceptual distance between two monochromatic stimuli scales non-linearly with the difference in wavelength (Fig. 5A, E), just as it is expected from choice experiments of similar colours^61–63^. In addition, we predict that perceptual distances are larger in the blue than in the yellow region of the spectrum (Fig. 5A), leading to better discrimination abilities in the former than in the latter^61,62^. Finally, both behavioural experiments^2^ and our modelling agree that perceptual distances are expected to be larger and discrimination better in between the peak sensitivities of receptors (~400 and ~500 nm) than at the peaks themselves (Fig. 5A).

Notably, when the same analysis was performed on the receptor sensitivity curves alone (Supplementary Fig. S7), or on a fully regular colour opponent model  (Fig. 5B), this yielded different predictions. Euclidean differences between receptor sensitivities did not increase monotonically with wavelength difference; we only find that once we have taken into account that receptors depolarize as a non-linear function of quantum catches (Supplementary Fig. S7). If we assume that colour is coded by two colour-opponent processes (in our example, along the axes of UV vs. blue-green and blue vs. UV-green, as suggested in^35^), this monotonic scaling disappears (Fig. 5F). This is important because only when the response difference vs. stimulus difference function is monotonic does perceptual distance reflect stimulus dissimilarity. Processing after the receptor level enhances the perceptual differences between similar colours in the random model, especially in the UV and blue region of the spectrum (Fig. 5C), implying that neural processing provides a boost in fine colour discrimination. In the regularly wired model, on the other hand, a boost is given to discriminating UV vs. blue and blue vs. green (Fig. 5D).

## Discussion

We propose that the diversity of colour-sensitive responses in the bee brain is best explained by assuming that these neurons receive randomly weighted inputs from all receptor types. The simplest and most parsimonious explanation for this type of neuronal organisation is the ease with which it can be implemented during neuronal development. A simple rule is sufficient to realise the connectivity pattern proposed by the model, where third-order visual neurons have a fixed morphology that defines the location, shape and target cell type of their branches, but where individual neurons make random connections with all target transmedullary cells they come in contact with during their growth. The presence of serotonin within the serpentine layer^38,56^ – a common neurotransmitter/neuromodulator that is often associated with synaptic plasticity – suggests the possibility that the random connections may be subsequently fine-tuned to stimuli in an experience dependent manner (as in^50,65^).

If the connections between receptors and colour sensitive neurons are indeed random, any given colour stimulus appearing at a given eye location will activate a different set of neurons in each bee; however, within one animal, it will consistently activate the same group of neurons (as in^46,66^). A direct consequence of the vast diversity of colour-sensitive neurons is that the responses of individual neurons are not comparable, and thus in themselves are insufficient to encode information. Instead, the ensemble response of the population^67^ of diverse colour opponent neurons may represent colour. Pooling the responses of a large set of colour-sensitive neurons could be potentially done by scanning an object (such as a flower) from a close distance; pooling a smaller set would result in coarser but more spatially accurate representation of colour. This requirement for active scanning could explain why restrained honeybees lose their ability to discriminate all but the most distinct colours^68^ and why the time needed to discriminate two coloured stimuli increases with colour similarity^69^. Finally, we identified an emergent structure in the population responses in our model, where perceptual distance increases with difference in input stimuli. Theory suggests that such a structure facilitates efficient learning, as decreased overlap of neural responses to a range of different stimuli can lead to sharpened neural tuning, thereby leading to improved discrimination^70^.

There is a tendency in neuroscience to associate specific neurons with specific tasks, describing a neuron’s response in terms of its most striking features (typically in terms of the stimuli that evoke the highest spike rates). This simplification has been called out before^71^ and is in conflict with our finding that one, morphologically uniform neuron type can generate diverse colour responses, without the need of assigning specific neuron types for specific colour coding tasks. From a machine learning point of view, there is no need for specific colour coding circuits; instead, state-of-the-art image recognition employs deep convolutional networks^72^. These networks are made up of neurons that exhibit learned and complex response properties that do not make sense in terms of features observed by humans.

Note that our model assumes that transmedullary cells, that connect the receptors and lamina fibres to third order cells, have the same spectral response properties as the receptors. Their known role in bees appears to be in the spatial summation of the signal (transmedullary cells send branches to neighbouring medulla columns in layers 1–2^38,51^) and in transforming the graded depolarisation signal to spike trains (tonic response) or spikes at the stimulus onset (phasic response)^38^. Recent advances indicate that in a different insect, *Drosophila melanogaster*, one element of colour-opponency processing could be implemented in the first stage of visual processing, through the mutual inhibition between two photoreceptors^73^. There is indirect evidence that inhibition exists among photoreceptors in bees^74^, and it is conceivable that lateral inhibition is present either among receptor terminals or transmedullary cells as well. If so, the columnar, retinotopically arranged transmedullary cells might already show colour opponency and other diverse wavelength dependent response; then, they will provide more varied input to third order cells than we assumed in the current model. Lateral inhibition between third order neurons, on the other hand, may offer an alternative to variable intrinsic neuronal properties^75^ as an explanation of thresholding effect in our model (as lateralization and thresholding of activation functions are theoretically analogous for the purpose of efficient neural representation^76–78^). Whether transmedullary cells do or do not alter the spectral properties of the signal will have to remain an open question until electrophysiological experiments explicitly test their spectral response profile.

In conclusion, our study adds to the growing body of data indicating randomness might be an important organizing principle in vision. We propose that random inputs, coupled with the lack of the characteristic spatial antagonism known for primates, explain the diversity of colour-sensitive responses in bees. This and similar insect vision studies are crucial for understanding the richness of visual processes, as they point us to novel and unexpected mechanisms by which visual processing is possible, deepening our understanding of neurobiology and providing material for useful applications such as in machine vision.

## Methods

### Receptor inputs to the model

The relative amount of light (quantum catch) absorbed by a particular type of photoreceptor is calculated as:

1 $$P=R{\int }_{300}^{700}{I}_{S}(\lambda )S(\lambda )D(\lambda )d\lambda $$

where *R* describes the overall sensitivity of the receptor, *I*_*S*_(*λ*) is the spectral reflectance function of the stimulus, *S*(*λ*) is the spectral sensitivity function of the receptor, and *D*(*λ*) is the illuminant^79,80^. Here, we set the sensitivity factor *R* = 6 for all photoreceptors and assumed perfectly uniform illumination (*D*(*λ*) = 1 at all *λ*). We used the the photoreceptor sensitivities of the honeybee from Peitsch *et al*.^5^.

The normalised (and so dimensionless) receptor response is directly calculated from the quantum catch *P*^79^:

2 $$E=P/(P+1)$$

It has been shown that the non-linearity (represented in Eq. 2) of the photoreceptor response is essential for understanding the responses of colour-sensitive neurons^35^. In our model, we consider *N* different sets of inputs *I* = (*I*^1^, *I*^2^, …*I*^*N*^). Then, the input *I*^*m*^(*m* = 1:*N*) is composed of responses of three different visual receptors ${I}^{m}=({E}_{b}^{m},{E}_{g}^{m},{E}_{uv}^{m})$. Here, ${E}_{b}^{m},{E}_{g}^{m},{E}_{uv}^{m}$ are the normalised responses of blue, green and UV receptors for the *m*^*th*^ colour input.

### Neural network model

In our model, three types of transmedullary cells ${R}^{m}=({r}_{1}^{m},\,{r}_{2}^{m},\,{r}_{3}^{m})$ are activated by colour inputs *I*^*m*^ through one-to-one connections (see Fig. 1), such that the change in output rate ${r}_{i}^{m}$ of these transmedullary cells are given by

3 $$\begin{array}{c}{\Delta }{r}_{1}^{m}={v}_{1}{E}_{b}^{m}\\ {\Delta }{r}_{2}^{m}={v}_{2}{E}_{g}^{m}\\ {\Delta }{r}_{3}^{m}={v}_{3}{E}_{uv}^{m}\end{array}$$

Here, *v*_i_ can take values between [−1; 0] and it is assumed to equal -1 (see Results for the inhibitory transmission hypothesis).

All synaptic weights, from transmedullary cells to the postsynaptic third order cells, are represented in a vector *W* = (*w*_1_, *w*_2_, *w*_3_). The total presynaptic input, *x*, to the postsynaptic third order cell *y* can be expressed as

4 $${x}^{m}=\sum _{i=1}^{3}{w}_{i}{\rm{\Delta }}{r}_{i}^{m}$$

Then, the firing rate change of a third-order cells is defined as:

5 $${\rm{\Delta }}{y}^{m}=F({x}^{m},\alpha )$$

where the *activation function*, *F*, is taken to be a saturating function of *x*, the total presynaptic input. The parameter *α* represents the sensitivity (i.e. the steepness) of the activation function.

**Figure 6:**
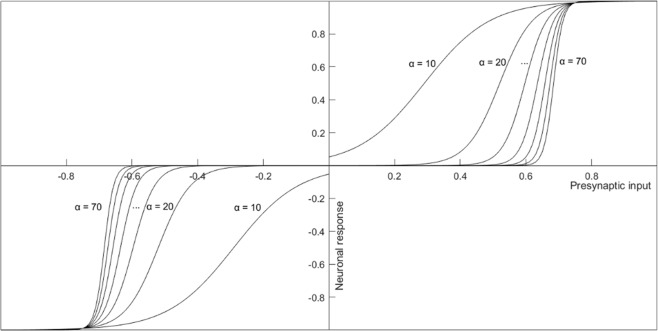
Activation functions of third-order neurons with different steepness (parameter α) in the firing rate change model. Curves are shown for α = 10; 20; 30; 40; 50; 60; 70. The shape of the activation function ensures that the modelled neurons do not respond at all below a certain threshold stimulation and respond maximally above another threshold. The neuron’s spectral response profile is defined by the presynaptic input weights and the α parameter of its activation function.

We define the activation function as a sigmoidal function that is symmetric for inhibitory and excitatory inputs:

6 $$F(x;\alpha )=\{\begin{array}{c}if\,x < 0:(\frac{-{A}_{0}}{1+{e}^{-\alpha (-x-b)}});\\ if\,x > 0:(\frac{{A}_{0}}{1+{e}^{-\alpha (x-b)}});\end{array}$$

where *A*_0_ is the maximum firing rate of the target cell (here, A_0_ = 1 for all neurons) and *α* controls the slope of the activation function (Fig. 6). The parameter *b* is the half-response sensitivity of the neuron. Aiming for comparability across neurons, we set *b* in a way that all neurons respond maximally at the same levels of input (i.e. F(x) = 0.99 at input x = 0.75):

7 $$b=\frac{ln\frac{1}{99}}{\alpha }+0.75$$

This basic version of the model calculates changes in firing rates, without making assumptions on the baseline firing activity of the neurons. We also report a modified version that can account for baseline and maximum firing rates and thus calculates actual firing rates. The results of the modified version are consistent with the firing rate change model (see Supplementary Fig. S3, S4).

### Fitting the model to empirical neurobiological data

For fitting to empirical neurophysiological data, we used the data from Kien and Menzel^28,30^ (see Results). The original papers only report relative changes of the firing rates, but no baseline or maximum firing rates. Accordingly, we used the firing rate change version of our model for finding the best fitting parameters. We have, however, added a possible firing rate representation as well (see Supplementary Fig. S3, S4). The parameters we fitted were the presynaptic weights to the third order neuron, the steepness of its activation function, and an extra parameter for the firing rate version of the model, the baseline firing rate. As our aim was to find the curves that match the overall shape of the empirical curves best, we added a 3x weighting factor to all peaks and troughs, and a 2x weighting factor to all no response measurements. When a neuron had different spectral tuning curves for phasic/tonic/off responses, those were treated as separate measurements. The input values and the best fit parameters can be found in Supplementary Tables S1, S2.

For each empirical spectral tuning curve, the best fit parameters of the model are iteratively estimated using a standard gradient descent algorithm under the least squares estimation method. We consider a set of *N* observations of the activities of third order cells *Y* = (*y*^1^, *y*^2^, …*y*^*N*^) that were evoked by different sets of colour inputs *I* = (*I*^1^, *I*^2^, …*I*^*N*^) respectively. We define the cost function, *G*, that must be minimized through an iteration process as:

8 $$G=\sum _{m=1}^{N}{({y}^{m}-\widehat{{y}^{m}})}^{2}$$

where $\widehat{{y}^{m}}$ represents the empirically measured response. Hence, we have

9 $$G=\sum _{m=1}^{N}{({y}^{m}-\widehat{{y}^{m}})}^{2}=\sum _{m=1}^{N}{(F(\sum _{i=1}^{3}{w}_{i}{r}_{i}^{m},\alpha )-\widehat{{y}^{m}})}^{2}$$

(*W*^*t*^, *α*^*t*^) represents the parameters already estimated in the previous iteration while (*W*^*t*+1^, *α*^*t*+1^) is updated via a standard gradient descent algorithm from (W^*t*^, *α*^*t*^). For the first iteration (t = 0), we use nominal initial parameters, *W*^*0*^ and *α*^*0*^.

An update of the vector of synaptic weights, *W*^*t*+1^, and the steepness of its activation function *α*^*t*+1^ are given as:

10 $$\begin{array}{c}{w}_{i}^{t+1}={w}_{i}^{t}-{\eta }_{w}\frac{\partial G}{\partial {w}_{i}}\\ {\alpha }^{t+1}={\alpha }^{t}-{\eta }_{\alpha }\frac{\partial G}{\partial \alpha }\end{array}$$

where *η*_*w*_ and *η*_*α*_ express the updating rate. Here, we used *η*_*w*_ = 0.001 and *η*_*α*_ = 0.001. The gradients are calculated as:

11 $$\begin{array}{c}\frac{{\rm{\partial }}G}{{\rm{\partial }}{w}_{i}}=\sum _{m=1}^{N}\,[2{r}_{i}^{m}{f}_{x}(\sum _{i=1}^{3}{w}_{i}{r}_{i}^{m},\alpha )(F(\sum _{i=1}^{3}{w}_{i}{r}_{i}^{m},\alpha )-\hat{{y}^{m}})]\\ \frac{{\rm{\partial }}G}{{\rm{\partial }}\alpha }=\sum _{m=1}^{N}\,[2{f}_{\alpha }(\sum _{i=1}^{3}{w}_{i}{r}_{i}^{m},\alpha )(F(\sum _{i=1}^{3}{w}_{i}{r}_{i}^{m},\alpha )-\hat{{y}^{m}})]\end{array}$$

here, ${f}_{x}=\frac{\partial F(x,\alpha )}{\partial x}$ and ${f}_{\alpha }=\frac{\partial F(x,\alpha )}{\partial \alpha }$ are the partial derivatives of the activation function respect to *x* and *α*.

The partial derivatives of the activation function *F*(*x*, *α*) given in the previous section are calculated as:

12 $$\begin{array}{c}{f}_{x}(x,\,\alpha )=\{\begin{array}{c}if\,x < 0:\frac{{A}_{0}\alpha {e}^{-\alpha (-x-b)}}{{(1+{e}^{-\alpha (-x-b)})}^{2}};\\ if\,x > 0:\frac{{A}_{0}\alpha {e}^{-\alpha (x-b)}}{{(1+{e}^{-\alpha (x-b)})}^{2}};\end{array}\\ {f}_{\alpha }(x,\,\alpha )=\{\begin{array}{c}if\,x < 0:\frac{-{A}_{0}(-x-b){e}^{-\alpha (-x-b)}}{{(1+{e}^{-\alpha (-x-b)})}^{2}};\\ if\,x > 0:\frac{{A}_{0}(x-b){e}^{-\alpha (x-b)}}{{(1+{e}^{-\alpha (x-b)})}^{2}};\end{array}\end{array}$$

The iteration is terminated either when the decrease in the cost function, *G*, approaches a plateau, or when increment of the vector norm of the parameters becomes smaller than an arbitrarily small $\varepsilon :\Vert {W}^{t+1}-{W}^{t}\Vert  < \varepsilon$. In our estimation, we iterated the algorithm 10^7^ times, which was enough to achieve these conditions.

### Spectral response characteristics of a population of randomly wired neurons

**Figure 7:**
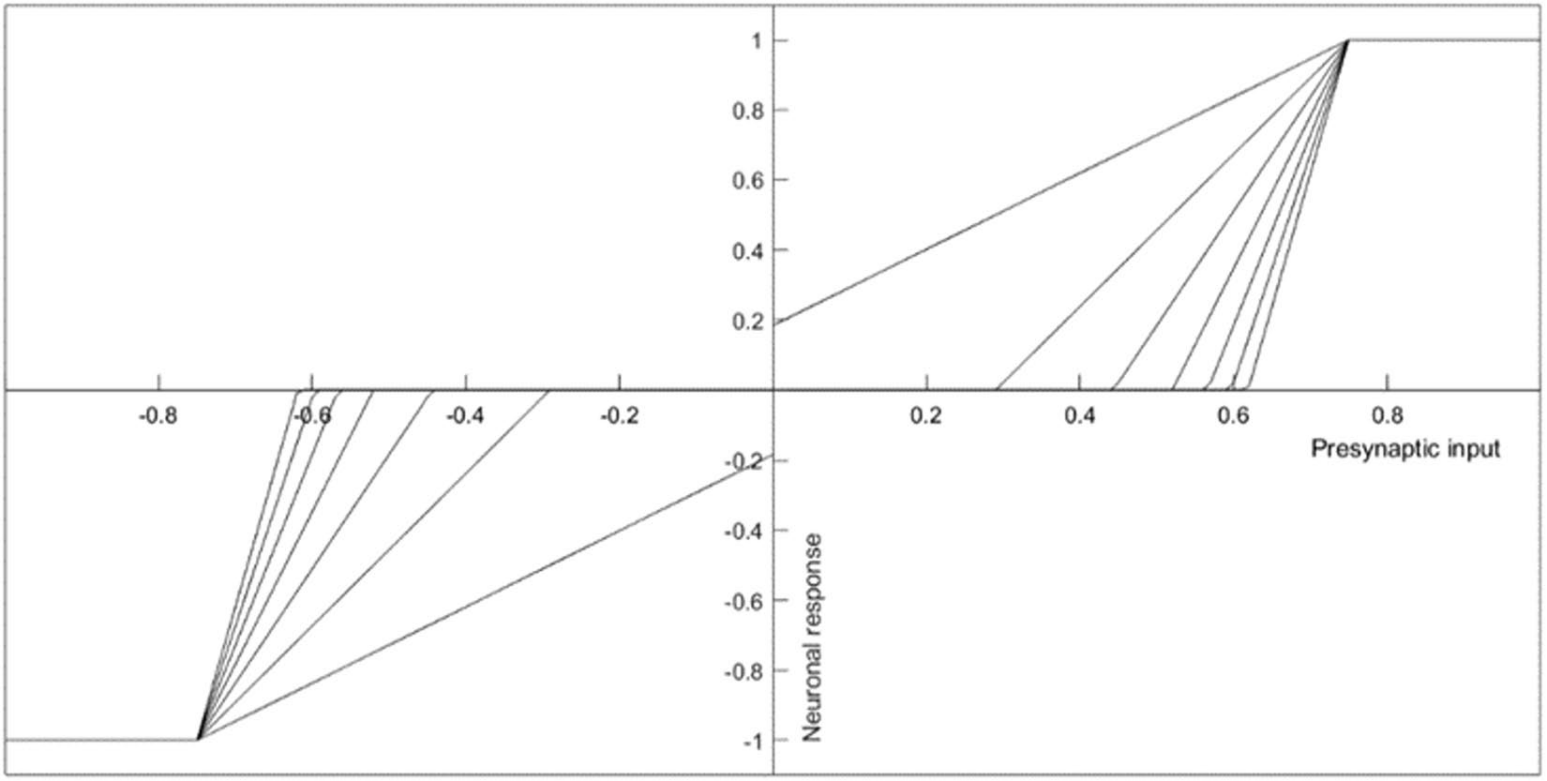
Simplified activation functions of third-order neurons with different steepness. The steepness values are chosen to match those on Fig. 6. Here, t_max_ = 0.75 and t_min_ = t_max_ −2(t_max_ − b) (i.e., the curves are defined by the maximum and the half response points).

For the exploration of the types of responses our model can produce, we approximated the sigmoidal response with a piecewise linear transfer function. The aim of the simplification was to speed up the calculations. Here, the *activation function*, $F$, is is defined as:

13 $${F}^{{\rm{^{\prime} }}}(x;\alpha )=\{\begin{array}{c}if\,x < -{t}_{max}:-\,1;\\ if-{t}_{max}\le x\le -\,{t}_{min}:-\,\frac{-x-{t}_{min}}{{t}_{max}-{t}_{min}};\\ \begin{array}{c}if-{t}_{min} < x < {t}_{min}:0;\\ if\,{t}_{min}\le x\le {t}_{max}:\frac{x-{t}_{min}}{{t}_{max}-{t}_{min}};\\ if\,x > {t}_{max}:1;\end{array}\end{array}$$

where the neuron does not respond under a minimum threshold *t*_*min*_, responds maximally above the maximum threshold *t*_*max*_; and responds linearly in between (Fig. 7).

For our third order neuron library, we assumed there exists one such neuron per medulla column and so generated 5500 randomly wired neurons. As an additional simplification, we set *t*_*max*_ = *max*(*x*_1_, *x*_2_…*x*_N_) for the *N* different sets of monochromatic inputs.

### Clustering analysis on empirical and simulated neuronal responses

To evaluate the optimal number of clusters that can best describe both the empirical weight distributions as well as the simulated neuronal responses, Dirichlet process with Gaussian mixture modelling^58^ and time-series K-Means analysis^81^ were performed using the scikit-learn^82^ and tslearn^59^ Python machine learning toolkits and with custom written Python scripts.

## Supplementary information

Supplemental Information

Dataset 2

Dataset 1

## Data Availability

All data generated or analysed during this study are included in this published article and its Supplementary Information Files. The source code for analysing the data during this study is available in the Github repository, https://github.com/FeiPengSMU/beeMedullaNeuronModel.
