# Supplementary material for: Randomly weighted receptor inputs can explain the large diversity of colour-coding neurons in the bee visual system
Source: Sci Rep. 2019 Jun 6;9:8330. doi: 10.1038/s41598-019-44375-0 (PMC6554269; doi:10.1038/s41598-019-44375-0)
Supplement: Supplementary file 1 — Supplemental Information [file 41598_2019_44375_MOESM1_ESM.docx]

**Randomly weighted receptor inputs can explain the large diversity of colour-coding neurons in the bee visual system**

Vera Vasas^1^, Fei Peng^2,*^, HaDi MaBouDi^1^, Lars Chittka^1,3^

^1^ Bee Sensory and Behavioural Ecology Lab, Department of Experimental and Biological Psychology, School of Biological and Chemical Sciences, Queen Mary, University of London, London, UK

^2^ Department of Psychology, School of Public Health, Southern Medical University, 1838 Guangzhou Road, Guangzhou 510515, Guangdong, China

^3^ Wissenschaftskolleg zu Berlin, Institute for Advanced Study, Wallotstrasse 19 D-14193, Berlin, Germany

^*^ Corresponding author [fpeng@smu.edu.cn]

**Supplementary Information**

***1. Spectral tuning curves of receptors and transmedullary cells in the model***

A


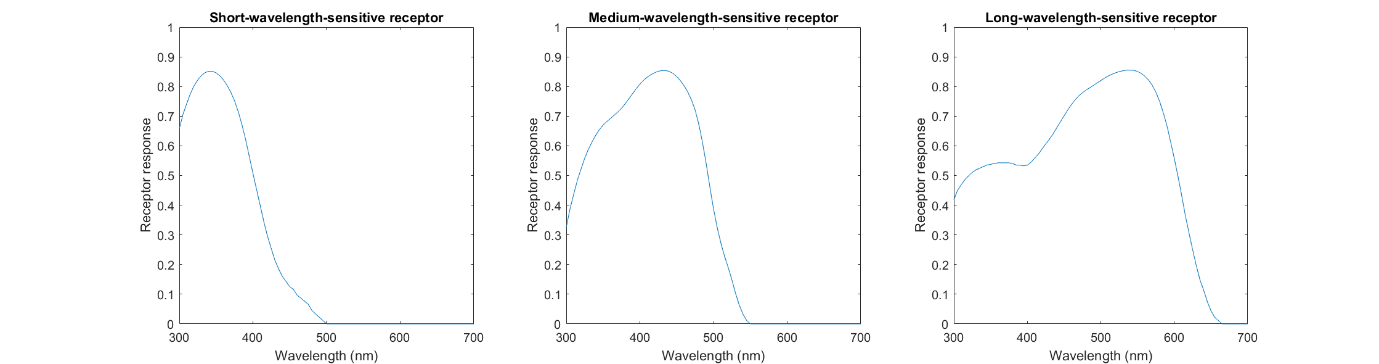

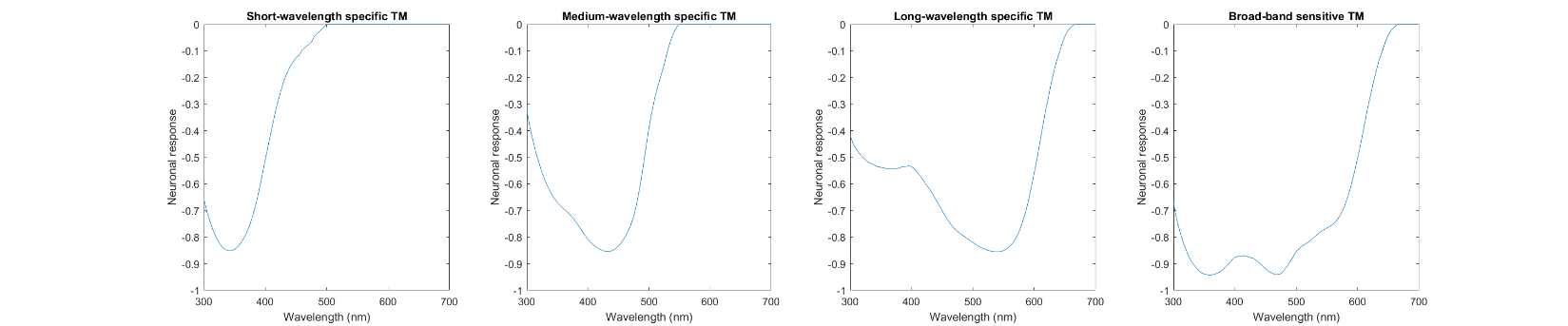


B

***Figure S1.*** *Spectral tuning curves of receptors and transmedullary cells. (A) Normalised (dimensionless) receptor responses of short-, medium- and long-wavelength-sensitive receptors to monochromatic lights of variable wavelength but equal intensity. (B) Normalised (dimensionless) responses of receptor-specific and broad-band transmedullary cells (TM) to monochromatic lights of variable wavelength but equal intensity, according to our model. A simple linear transfer (but with a reverse sign) is assumed between receptors and receptor-specific transmedullary cells; broad-band transmedullary neurons receive input from all receptor types (weighting factors for the example shown are v_1_=-0.3, v_2_=-0.3, v_3_=-0.9).*

***2. Spectral tuning curves of model neurons fitted to empirical neurobiological data***


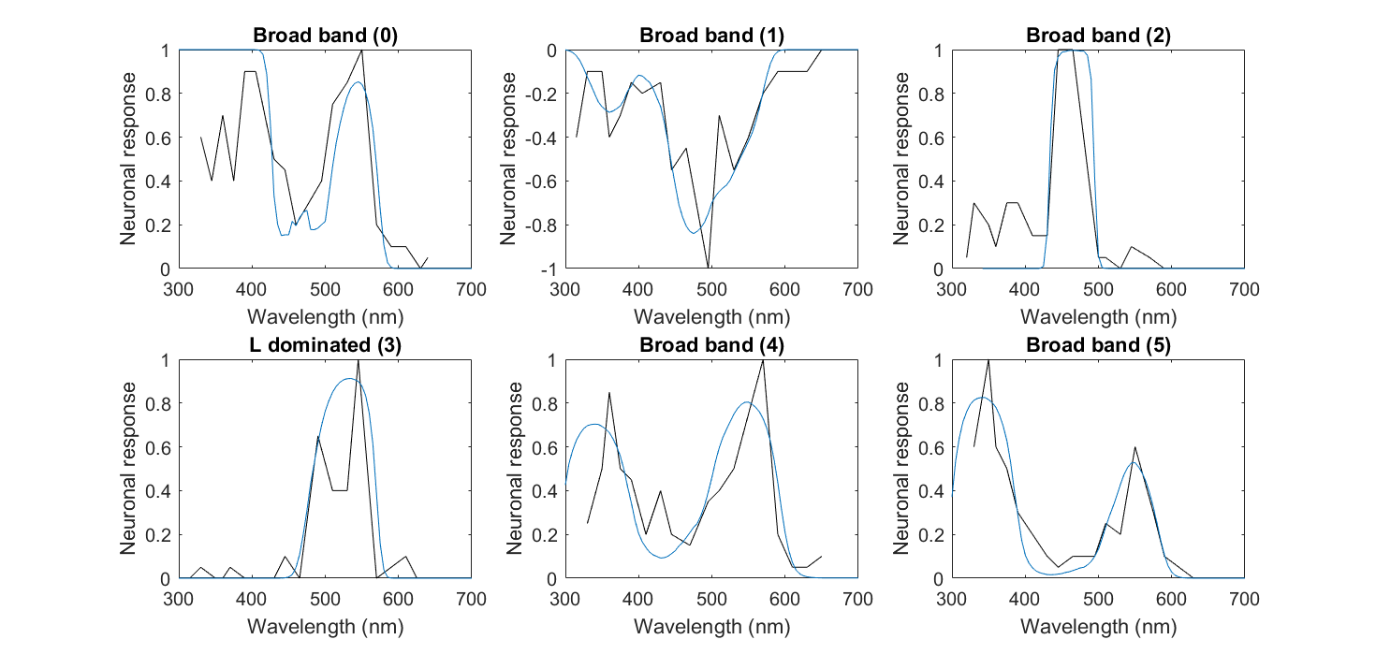

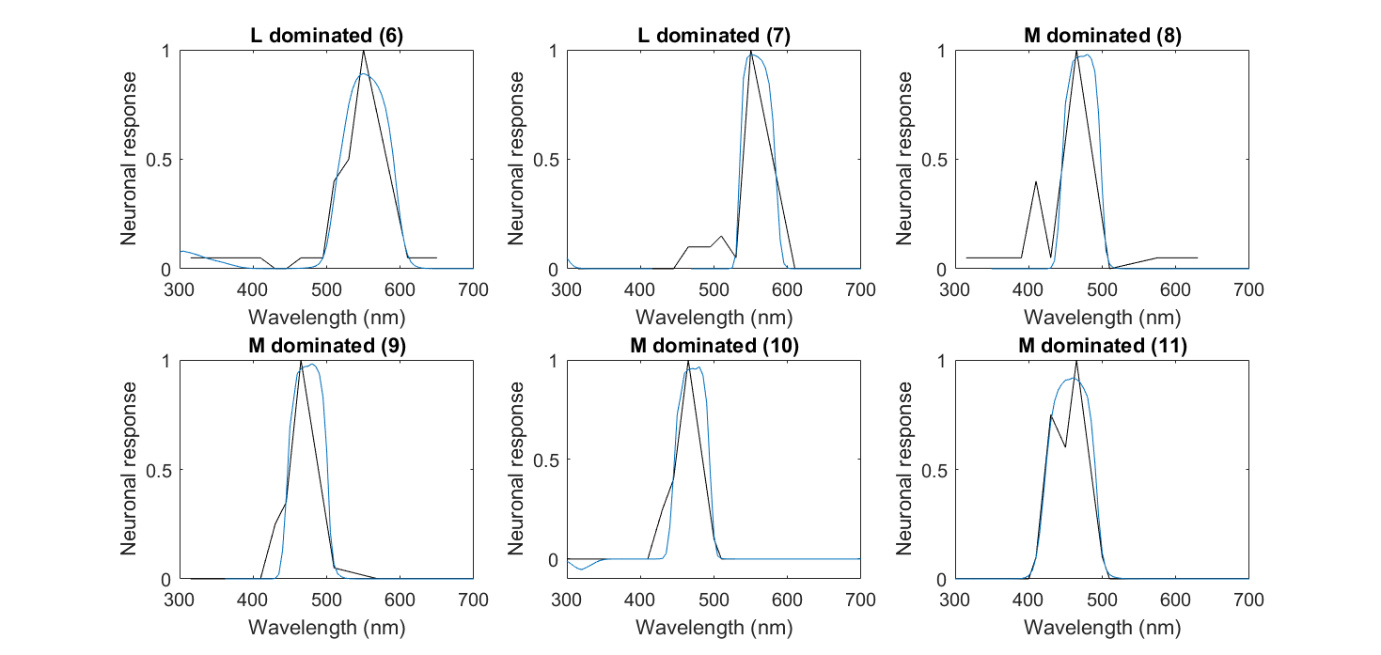


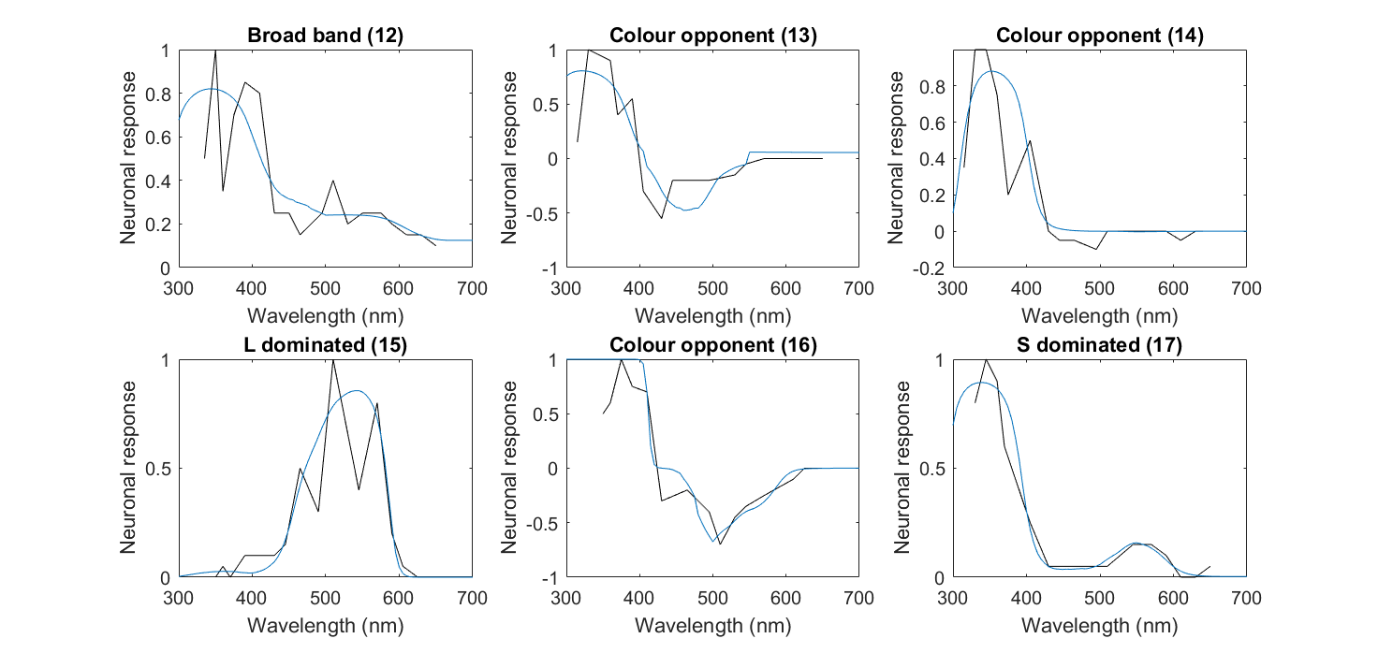

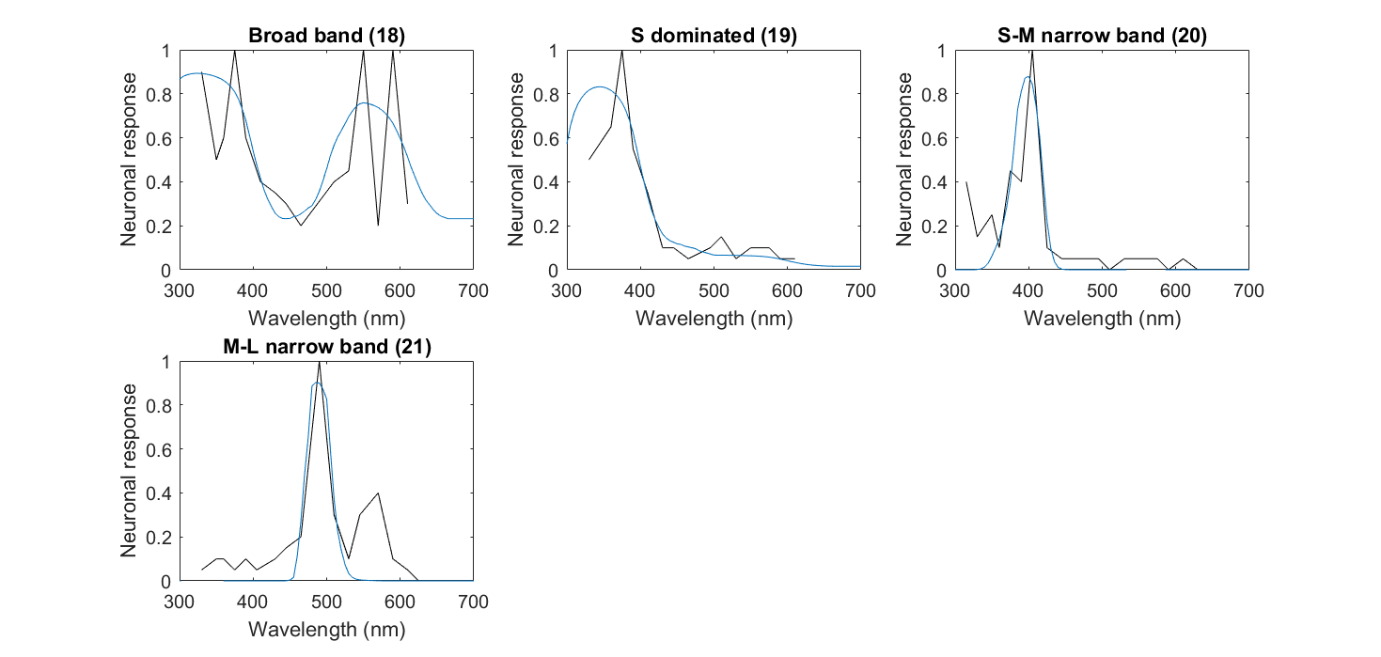


***Figure S2.*** *Best fit spectral tuning curves produced by the firing rate change model. All figures show the normalised (dimensionless) change in neuron responses as a function of monochromatic lights of variable wavelength but equal intensity. Black lines depict the empirically measured spectral tuning curves; blue lines indicate the best fit curves generated by the model.*

| **index** | **α** | **w_1_** | **w_2_** | **w_3_** | **R^2^** |
| --- | --- | --- | --- | --- | --- |
| 0 | 51.42 | -0.92 | 0.08 | -0.81 | 0.86 |
| 1 | 36.14 | 0.14 | 0.13 | 0.72 | 0.86 |
| 2 | 45.39 | 1.35 | -0.86 | -0.28 | 0.93 |
| 3 | 68.59 | 0.07 | -0.03 | -0.83 | 0.80 |
| 4 | 15.58 | -0.35 | 0.20 | -0.64 | 0.88 |
| 5 | 21.13 | -0.45 | 0.18 | -0.63 | 0.94 |
| 6 | 17.02 | -0.33 | 0.55 | -0.71 | 0.96 |
| 7 | 40.16 | -1.03 | 1.49 | -0.86 | 0.99 |
| 8 | 45.73 | 1.31 | -0.61 | -0.47 | 0.94 |
| 9 | 44.69 | 1.36 | -0.58 | -0.51 | 0.98 |
| 10 | 46.27 | 1.42 | -0.69 | -0.39 | 0.98 |
| 11 | 23.41 | 0.55 | -0.66 | -0.24 | 0.97 |
| 12 | 8.72 | -0.39 | -0.01 | -0.11 | 0.90 |
| 13 | 9.90 | -0.82 | 0.45 | -0.01 | 0.77 |
| 14 | 19.08 | -0.52 | -0.44 | 0.24 | 0.89 |
| 15 | 23.47 | -0.02 | 0.03 | -0.74 | 0.88 |
| 16 | 15.72 | -2.35 | 0.22 | 0.50 | 0.90 |
| 17 | 13.70 | -0.58 | 0.17 | -0.34 | 0.98 |
| 18 | 7.72 | -0.56 | 0.39 | -0.36 | 0.89 |
| 19 | 11.61 | -0.47 | -0.02 | -0.14 | 0.92 |
| 20 | 52.96 | -0.23 | -1.16 | 0.68 | 0.86 |
| 21 | 61.81 | 1.36 | -0.34 | -0.68 | 0.89 |

***Table S1.*** *Best fit parameters proposed by the firing rate change model for the empirical neurobiological measurements from ^1,2^.*

***3. Firing rate model***

Here, we explicitly represent firing rates (and not only the changes in firing rates as before). Thus, the output rate $r_{i}^{m}$ of three types of the transmedullary cells are given by

| $r_{1}^{m}=V_{0}+v_{1}E_{b}^{m}$  $r_{2}^{m}=V_{0}+v_{2}E_{g}^{m}$  $r_{3}^{m}=V_{0}+v_{3}E_{uv}^{m}$ | (1) |
| --- | --- |

where $V_{0}$ is the averaged base-line activity of transmedullary cells. Here $V_{0}=1$, and, as before, $v_{i}$ can take values between$\left[ -1;0 \right]$ and it is assumed to equal -1.

Again, all synaptic weights from transmedullary cells to the third-order cells are represented in a vector $W=\left( w_{1}, w_{2}, w_{3} \right)$. The total presynaptic input to the postsynaptic third-order cell $y^{m}$ can be expressed as

| $x^{'}=\sum_{i=1}^{3} w_{i}r_{i}^{m}+C$ | (2) |
| --- | --- |

where $C$ represents a modifier to the neuron’s baseline firing rate. Then, the firing rate is defined as

| $y^{m}=F\left( x', \alpha,\beta\right)$ | (3) |
| --- | --- |

The baseline firing rate can then be calculated as $y^{m}$ without presynaptic inputs. The parameter $\alpha$ controls the sensitivity of the postsynaptic neuron to its inputs, and $\beta$ defines the half-response point of the neuron without the baseline. Here, we set $\beta=0$ .

We define the activation function as

| $F\left( x';\alpha,\beta\right)= \frac{A_{0}}{1+e^{-\alpha(x'-\beta)}}$ | (4) |
| --- | --- |


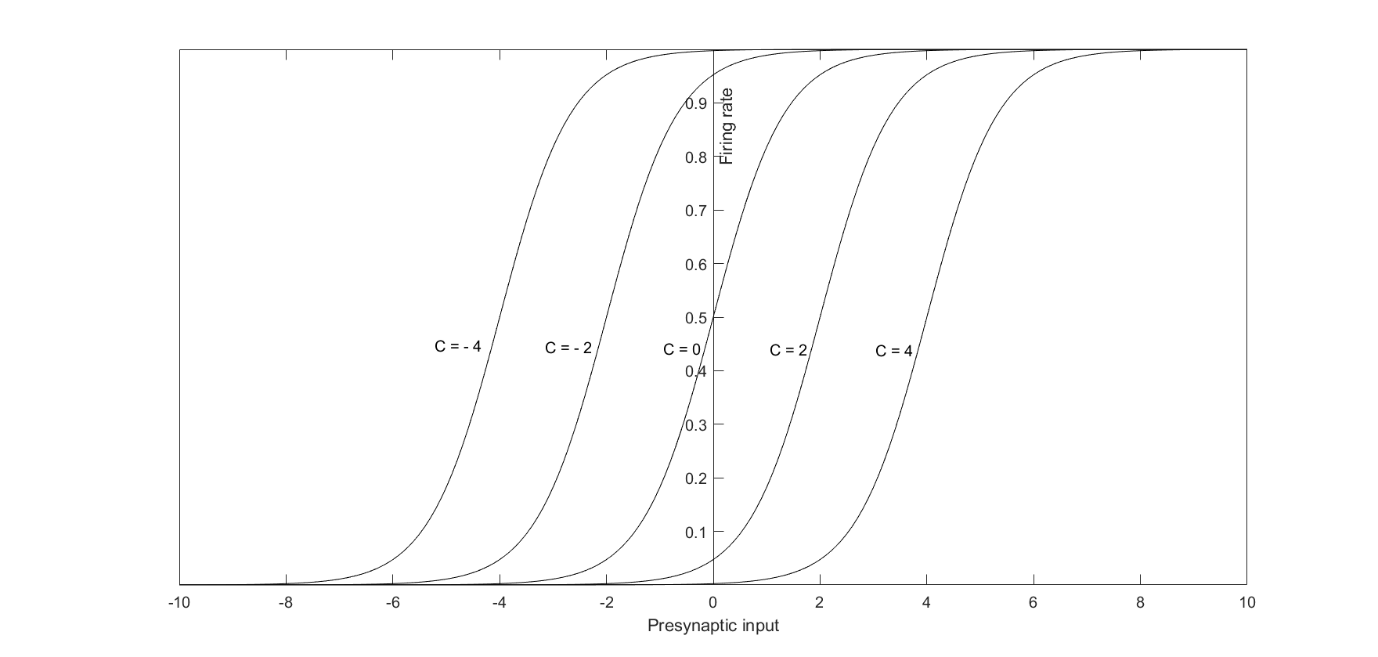


***Figure S3.*** *Activation functions of third order neurons in the firing rate model with variable baseline firing rates. Here, β = 0 and α = 1.5. Curves are shown for C = -4; -2; 0; 2; 4.*

***4. Fitting the firing rate model to the empirical data***

The original data do not include baseline and maximum firing rates, only relative changes to the neuron’s firing rate^1,2^. Here we made the assumption that the neuron’s firing rate is zero at the minimum of the spectral tuning curve and it is one at the maximum of the curve. This assumption, although arbitrary, allowed us to show how the model can reproduce firing rate data.

Fitting the firing rate model to the modified empirical data follows the same logic as to the firing rate change model. Here, the cost function is defined as

| $G=\sum_{m=1}^{N} \left( y^{m}-\hat{y^{m}} \right)^{2}=\sum_{m=1}^{N} \left( F\left( \sum_{i=1}^{3} w_{i}r_{i}^{m}+C, \alpha,\beta\right)-\hat{y^{m}} \right)^{2}$ | (5) |
| --- | --- |

$\theta^{t}=\left( W^{t}, \alpha^{t}, C^{t} \right)$ represents the parameters already estimated in the previous iteration while $\theta^{t+1}=\left( W^{t+1}, \alpha^{t+1}, C^{t+1} \right)$ is updated via a gradient algorithm from $\theta^{t}$. For the first iteration ($t=0$), we use a nominal initial parameter, $\theta^{0}$.

The update of the vector of synaptic weights, $\theta^{t+1}$, is given as

| $w_{i}^{t+1}=w_{i}^{t}-\eta_{w}\frac{\partial G}{\partial w_{i}}$  $\alpha^{t+1}=\alpha^{t}-\eta_{\alpha}\frac{\partial G}{\partial\alpha}$  $C^{t+1}=C^{t}-\eta_{C}\frac{\partial G}{\partial C}$ | (6) |
| --- | --- |

where $\eta_{w}, \eta_{C}$ and $\eta_{\alpha}$express the updating rates. Here, we used $\eta_{w}=0.7$, and $\eta_{\alpha}=0.001$ and $\eta_{C}=0.001$. The gradient is calculated as

| $\frac{\partial G}{\partial w_{i}}=\sum_{m=1}^{N} \left[ 2r_{i}^{m}f_{x}\left( \sum_{i=1}^{3} w_{i}r_{i}^{m}+C, \alpha, \beta\right) \left( F\left( \sum_{i=1}^{3} w_{i}r_{i}^{m}+C, \alpha,\beta\right)-\hat{y^{m}} \right) \right]$  $\frac{\partial G}{\partial\alpha}=\sum_{m=1}^{N} \left[ 2f_{\alpha}\left( \sum_{i=1}^{3} w_{i}r_{i}^{m}+C, \alpha,\beta\right) \left( F\left( \sum_{i=1}^{3} w_{i}r_{i}^{m}+C, \alpha,\beta\right)-\hat{y^{m}} \right) \right]$  $\frac{\partial G}{\partial C}=\sum_{m=1}^{N} \left[ 2f_{x}\left( \sum_{i=1}^{3} w_{i}r_{i}^{m}+C, \alpha,\beta\right) \left( F\left( \sum_{i=1}^{3} w_{i}r_{i}^{m}+C, \alpha,\beta\right)-\hat{y^{m}} \right) \right]$ | (7) |
| --- | --- |

here, $f_{x}=\frac{\partial F\left( x, \alpha\right)}{\partial x}$ and $f_{\alpha}=\frac{\partial F\left( x, \alpha\right)}{\partial\alpha}$ are the partial differentials of the activation function, and they are given as

| $f_{x}\left( x;\alpha,\beta\right)= \frac{A_{0}\alpha e^{-\alpha(x-\beta)}}{\left( 1+e^{-\alpha(x-\beta)} \right)^{2}}$  $f_{\alpha}\left( x;\alpha,\beta\right)= \frac{A_{0}\left( x-\beta\right)e^{-\alpha(x-\beta)}}{\left( 1+e^{-\alpha(x-\beta)} \right)^{2}}$ | (8) |
| --- | --- |

The iteration is terminated either when decrease in the cost function,$G,$ approaches a plateau, or when increment of the vector norm of the parameters becomes smaller than$\varepsilon$: $\|\theta^{t+1}-\theta^{t}\|<\varepsilon$. These criteria are achieved after 40000 iterations.

**
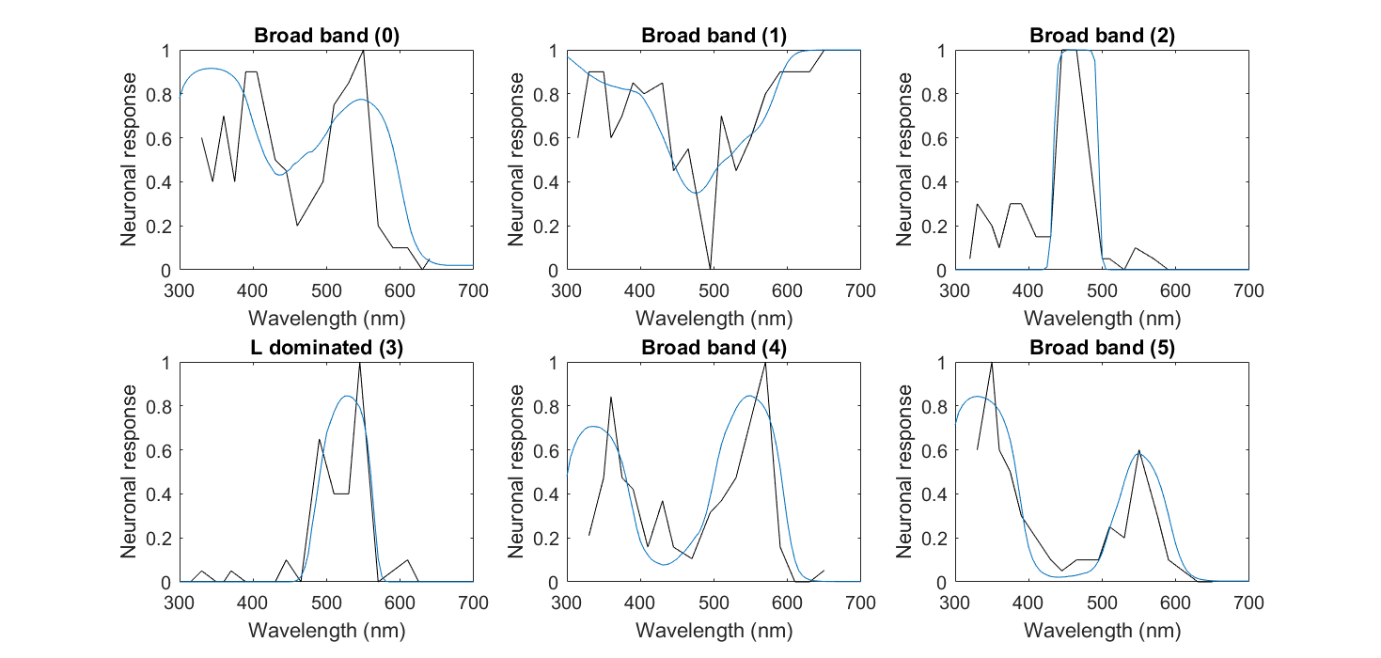
**

**
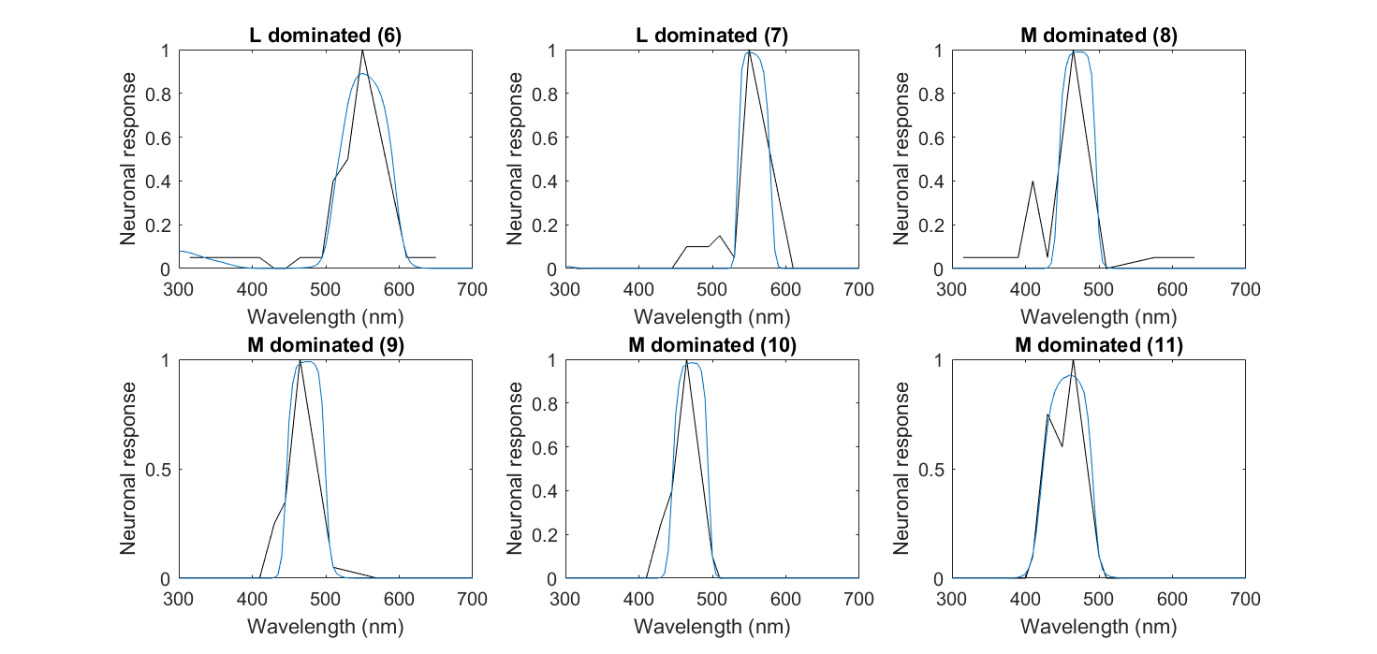
**

**
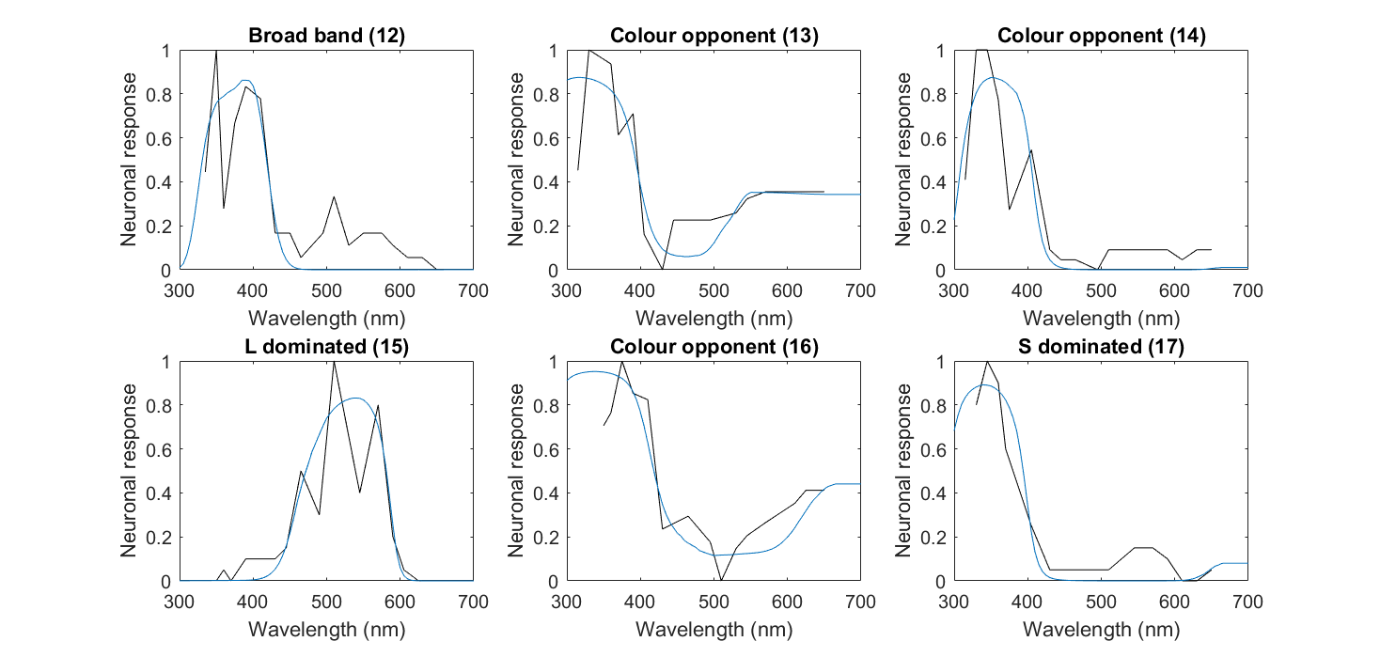
**

**
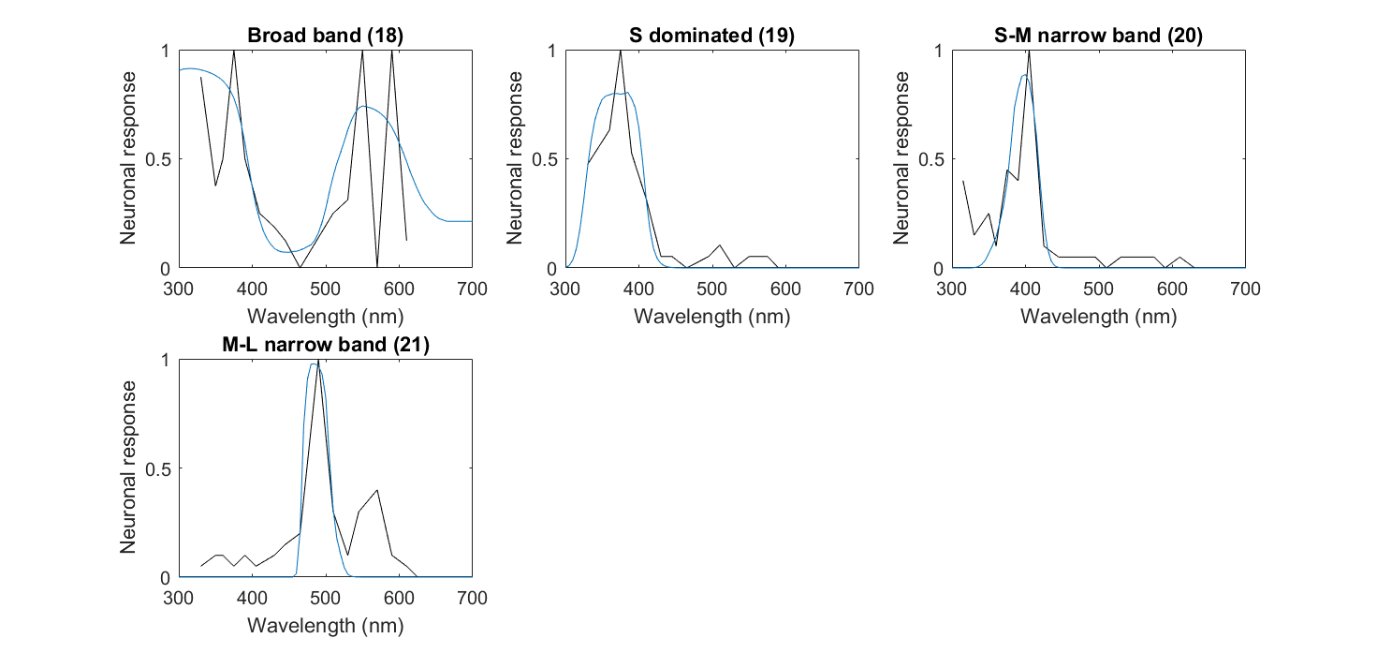
**

***Figure S4.*** *Best fit spectral tuning curves produced by the modified firing rate model. All figures show the normalised (dimensionless) change in neuron response as a function of the wavelength of monochromatic lights of equal intensity. Black lines depict the empirically measured spectral tuning curves; blue lines indicate the best fit curves generated by the model.*

| **index** | **α** | **w_1_** | **w_2_** | **w_3_** | **C** |
| --- | --- | --- | --- | --- | --- |
| 0 | 1.07 | -4.27 | 1.25 | -5.59 | 4.98 |
| 1 | 1.27 | -0.17 | 1.83 | 6.69 | -2.27 |
| 2 | 1.93 | 18.49 | -24.56 | -31.38 | -0.02 |
| 3 | 1.76 | 9.25 | -2.61 | -42.00 | 0.12 |
| 4 | 1.29 | -4.26 | 2.91 | -7.61 | 3.80 |
| 5 | 1.37 | -5.70 | 3.58 | -5.12 | 3.12 |
| 6 | 1.13 | -4.90 | 8.35 | -10.67 | -0.01 |
| 7 | 2.10 | -26.14 | 32.21 | -26.17 | -0.09 |
| 8 | 1.87 | 15.72 | -17.89 | -34.30 | -0.02 |
| 9 | 1.83 | 15.14 | -15.46 | -34.62 | -0.02 |
| 10 | 1.84 | 15.37 | -18.02 | -33.63 | -0.01 |
| 11 | 1.21 | 7.91 | -13.60 | -9.41 | -0.01 |
| 12 | 1.28 | -5.37 | -15.03 | 14.10 | 0.08 |
| 13 | 0.99 | -5.37 | 3.34 | -0.05 | 1.42 |
| 14 | 1.16 | -7.52 | -7.70 | 11.19 | 0.14 |
| 15 | 1.13 | 3.29 | 0.05 | -13.82 | 0.05 |
| 16 | 1.24 | -4.27 | 0.31 | 1.61 | 2.16 |
| 17 | 1.09 | -8.49 | -1.49 | 7.63 | 0.12 |
| 18 | 1.22 | -4.83 | 3.89 | -2.27 | 2.14 |
| 19 | 1.35 | -8.48 | -16.02 | 15.65 | 0.17 |
| 20 | 1.81 | -6.06 | -32.53 | 22.22 | -0.21 |
| 21 | 2.90 | 20.71 | -13.79 | -67.60 | -0.13 |

***Table S2.*** *Best fit parameters proposed by the modified firing rate model for the edited empirical neurobiological measurements from ^1,2^.*

***5. Clustering of model weights***


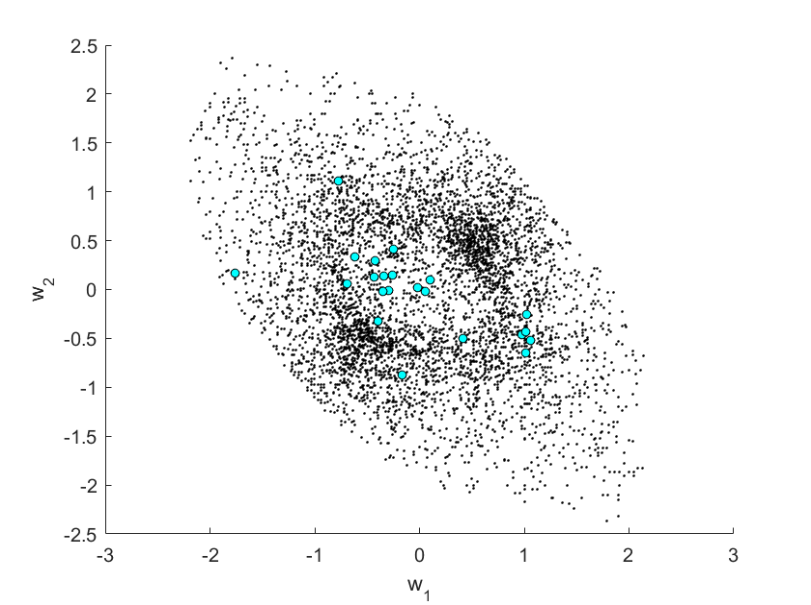

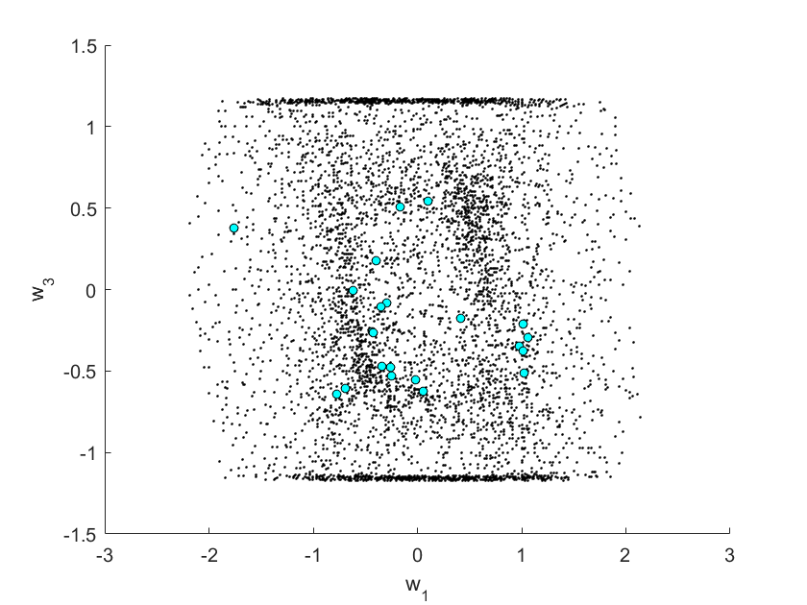

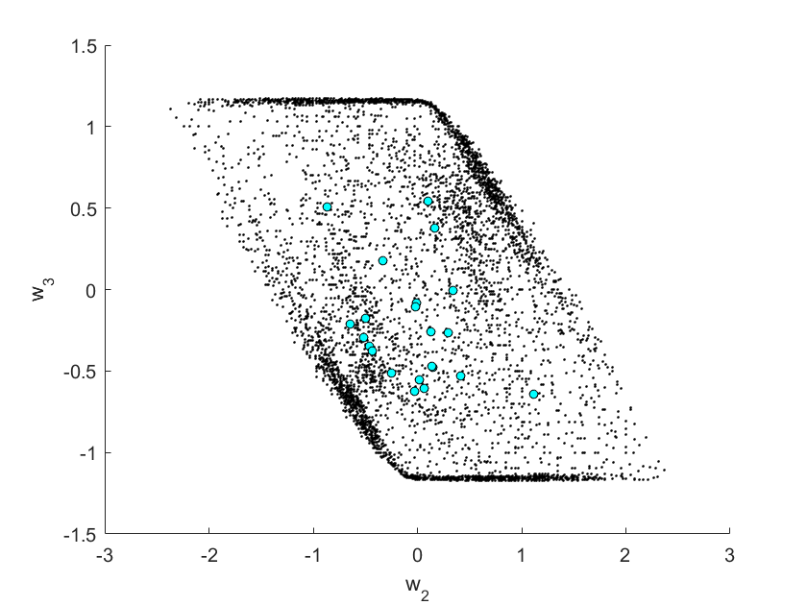


***Figure S5.*** *Weight distributions. Blue dots indicate weights fitted to empirical data, black dots indicate randomly chosen weights in our library of 5500 neurons. Both set of weights are normalised to give maximum response (*$y=1$*) at unit presynaptic input* $x =1$*.*

***6. Clustering of model response curves***

***
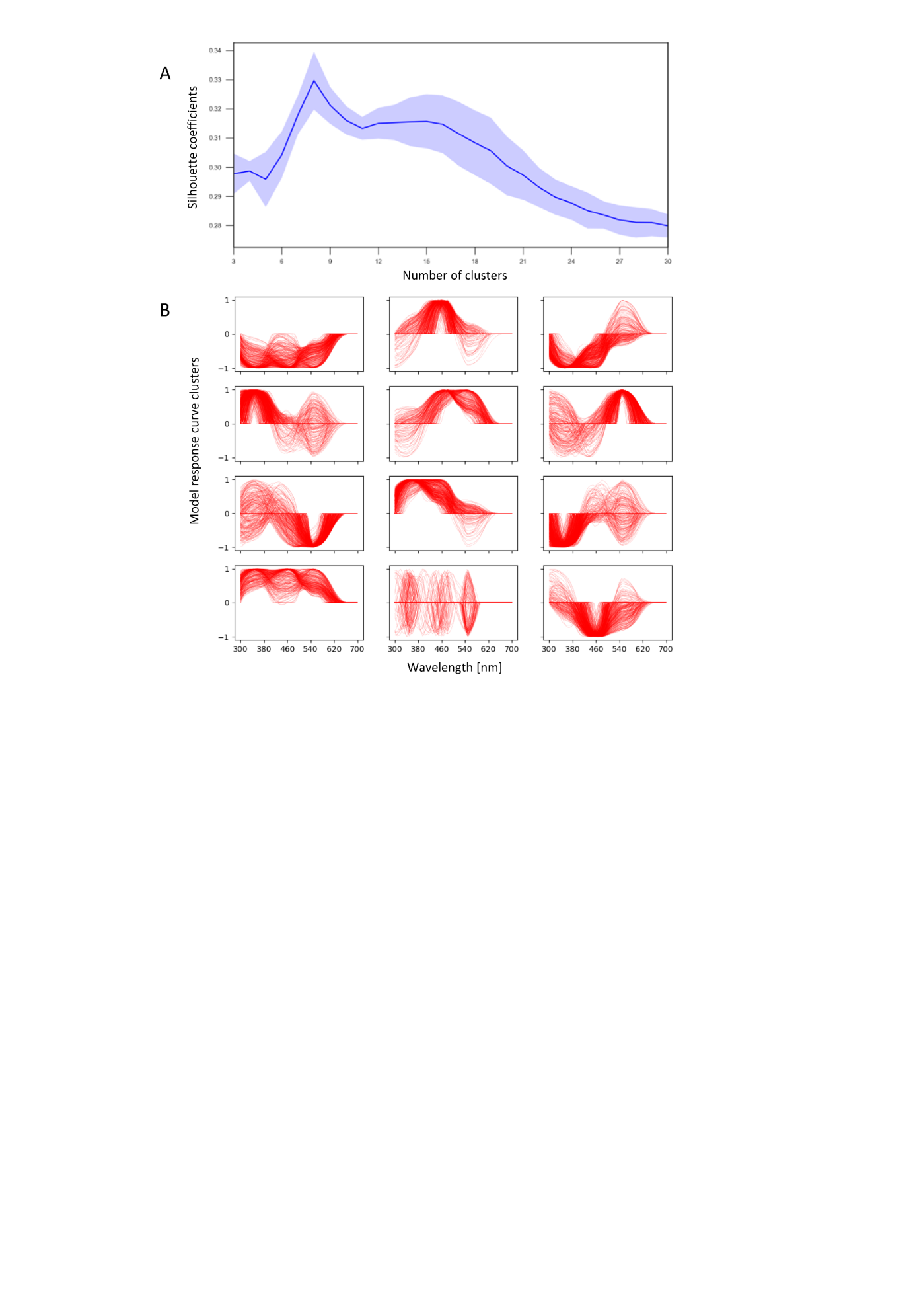
***

***Figure S6.*** *Additional clustering analysis on model response curves. A. Through time-series K-Means clustering ^3,4^ together with silhouette analysis ^5^, we show that the 5500 neuronal responses could be best described by 8-16 clusters (mean ±SD on silhouette coefficients for each cluster number shown, repeated for 100 times). B. Spectral response curves grouped by time-series K-Means clustering into 12 clusters are plotted as 12 panels.*

***7. Perceptual distances***


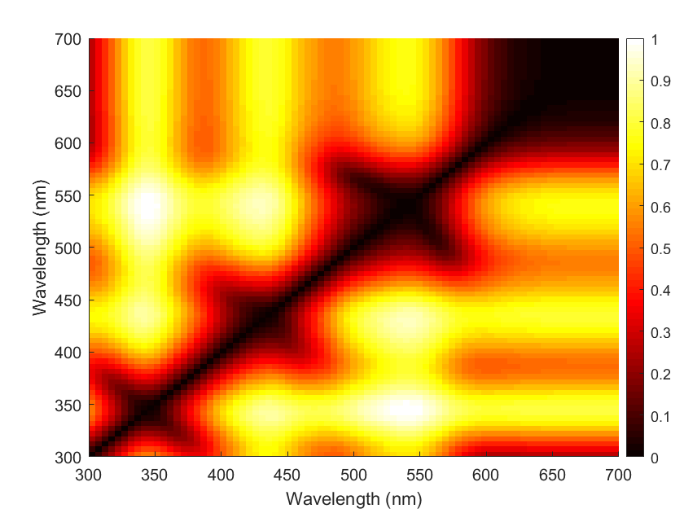

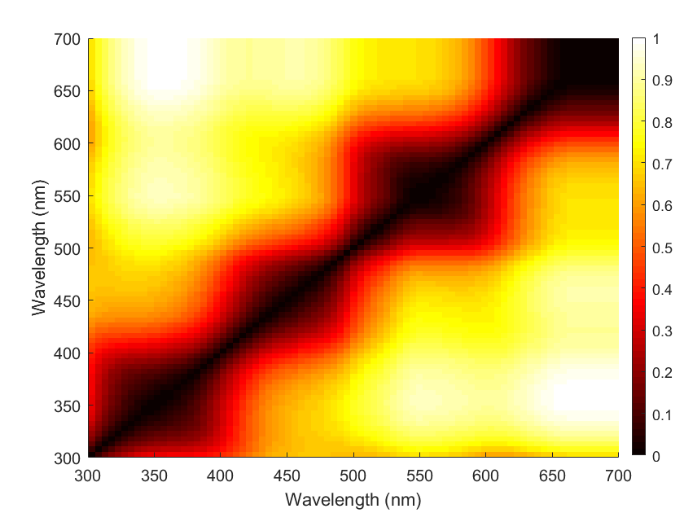


B

A

***Figure S7.***  *(A) Normalised Euclidian distances across receptor sensitivities at different wavelengths across the spectrum visible for bees. (B) Normalised Euclidian distances across receptor responses to all combinations of monochromatic stimuli.*

***References***

1. Kien, J. & Menzel, R. Chromatic properties of interneurons in the optic lobes of the bee - I. broad band neurons. *J. Comp. Physiol.* **113,** 17–34 (1977).

2. Kien, J. & Menzel, R. Chromatic properties of interneurons in the optic lobes of the bee - II. narrow band and colour opponent neurons. *J. Comp. Physiol.* **113,** 35–53 (1977).

3. Tavenard, R. tslearn: A machine learning toolkit dedicated to time-series data. (2017).

4. Huang, X. *et al.* Time series k-means: A new k-means type smooth subspace clustering for time series data. *Inf. Sci. (Ny).* **367–368,** 1–13 (2016).

5. Rousseeuw, P. J. Silhouettes: A graphical aid to the interpretation and validation of cluster analysis. *J. Comput. Appl. Math.* **20,** 53–65 (1987).
